# SOX17-positive rete testis epithelium is required for Sertoli valve formation and normal spermiogenesis in the male mouse

**DOI:** 10.1038/s41467-022-35465-1

**Published:** 2022-12-21

**Authors:** Aya Uchida, Kenya Imaimatsu, Honoka Suzuki, Xiao Han, Hiroki Ushioda, Mami Uemura, Kasane Imura-Kishi, Ryuji Hiramatsu, Hinako M. Takase, Yoshikazu Hirate, Atsuo Ogura, Masami Kanai-Azuma, Akihiko Kudo, Yoshiakira Kanai

**Affiliations:** 1 Department of Veterinary Anatomy, The University of Tokyo, Bunkyo-ku, Tokyo Japan; 2 Bioresource Engineering Division, RIKEN BioResouce Research Center, Tsukuba, Ibaraki Japan; 3 Center for Experimental Animals, Tokyo Medical and Dental University, Bunkyo-ku, Tokyo Japan; 4 Department of Microscopic Anatomy, Kyorin University School of Medicine, Mitaka, Tokyo Japan

**Keywords:** Urinary tract, Morphogenesis, Reproductive biology

## Abstract

Seminiferous tubules (STs) in the mammalian testes are connected to the rete testis (RT) via a Sertoli valve (SV). Spermatozoa produced in the STs are released into the tubular luminal fluid and passively transported through the SV into the RT. However, the physiological functions of the RT and SV remain unclear. Here, we identified the expression of *Sox17* in RT epithelia. The SV valve was disrupted before puberty in RT-specific *Sox17* conditional knockout (*Sox17-*cKO) male mice. This induced a backflow of RT fluid into the STs, which caused aberrant detachment of immature spermatids. RT of *Sox17-*cKO mice had reduced expression levels of various growth factor genes, which presumably support SV formation. When transplanted next to the *Sox17*^+^ RT, Sertoli cells of *Sox17-*cKO mice reconstructed the SV and supported proper spermiogenesis in the STs. This study highlights the novel and unexpected modulatory roles of the RT in SV valve formation and spermatogenesis in mouse testes, as a downstream action of *Sox17*.

## Abstract (web summary)

A valve-like structure called this Sertoli valve (SV) supports spermatogenesis by modulating the directional fluid flow in mouse testis. The SV formation is supported by its neighboring SOX17 + rete testis (RT). This study highlights the essential roles of RT and SV in spermatogenesis.

## Introduction

A pool of undifferentiated spermatogonia (A_undiff_) resides within the basal compartment of the convoluted seminiferous tubules (STs) in mammalian testes, supporting continuous spermatogenesis throughout adulthood^1^. A_undiff_ population encompasses Spermatogonial stem cells (SSCs) as a subpopulation. Self-renewal of the SSCs is supported in the niche that serves a growth factor milieu, represented as Glial cell line-derived neurotrophic factor (GDNF) and fibroblast growth factors (FGFs)^2–5^. Upon exposure to retinoic acid (RA), differentiating spermatogonia (A_diff_) are recruited from the A_undiff_ pool^6–9^. A_diff_ then differentiates into preleptotene spermatocytes to initiate meiosis while translocating into the adluminal compartment of the ST^9–11^. The initiation of meiosis is mediated by RA which upregulates *Stimulated by retinoic acid gene 8 (Stra8)* expression^12^. After completing meiosis, haploid round spermatids differentiate into elongated spermatids that subsequently undergo final morphological differentiation into spermatozoa (*i.e*., spermiogenesis)^13^. While developing spermatids are tightly anchored to the apical surfaces of Sertoli cells, fully differentiated spermatozoa are released into the lumen of the ST (*i.e*., spermiation) upon exposure to RA, and are passively transported by the luminal fluid towards the RT^9,13^.

In various vertebrate species including mammals, the RT serves as a channel for sperm transport from the ST to the efferent duct^14,15^. Its epithelium consists of a single layer of cuboidal epithelial cells with microvilli and primary cilium on the apical surface in mice. The RT epithelium expresses markers for gonadal somatic cells such as SRY-Box transcription factor 9 (SOX9), GATA binding protein 4 (GATA4), and nuclear receptor subfamily 5 group A member 1 (NR5A1; also known as SF1)^16–20^. Whereas it is uniquely distinguished by the ubiquitous expression of paired box 8 (PAX8) and cadherin-1 (CDH1), together with its partial expression of keratin-8 (KRT8)^16–20^. Recent studies have started shedding light on the embryonic development of RT^16,19,20^. However, apart from its well-appreciated roles in sperm transport, little is known about the contributions of the RT to testicular structure and function in male sexual maturation.

A valve is a structure conventionally found in the cardiovascular and lymphatic circulatory systems^8^. It passively creates unidirectional flow in the channel, thus preventing retrograde flow in the system^21,22^. Deformation of a valve affects the channel geometry, which in turn decreases intraluminal pressure, causing valve regurgitation^8^. As an organ with active oscillatory fluid flow^23^, the mammalian testis forms a valve-like structure called the Sertoli valve (SV) at the terminal end of each ST around the peripubertal period^24–27^. The SV is constructed by ‘modified’ Sertoli cells with constitutively high expression of phosphorylated AKT (p-AKT); cytoplasmic processes filled with acetylated tubulin-positive (ace-TUB^+^) microtubular bundles protrude toward the RT lumen to form a valve-like structure^24–27^. Intriguingly, the SV epithelia serve as an anatomical niche for A_undiff_^24^. This process is mediated by local repression of RA-induced germ cell differentiation and constitutively high expression of mitogens for A_undiff_, supporting their self-renewal (*i.e*., FGFs and GDNF)^2–5,25^. The Sertoli cells constituting the SV are non-cell-autonomously specified by the adjacent RT^25^. This suggests a paracrine-related mechanism in the SV epithelia involving the adjacent RT. Based on its morphology, the SV may prevent the reflux of seminiferous tubular fluid and spermatozoa from the RT back to the ST^28,29^. Although the SV and RT are phylogenetically conserved structures across mammalian species^26^, the physical and physiological roles of the RT and the SV remain largely unknown.

In this study, we identified the expression of *SRY-delated HMG box factor-17 (Sox17)* in RT epithelial cells and demonstrated its crucial roles in the formation of the SV and spermiogenesis in upstream STs. This study provides the first direct evidence that the RT orchestrates spermatogenesis in the ST, and sheds further light on the indispensable role of the SV in the testicular fluid dynamics of the testes.

## Results

### SOX17 expression in the RT epithelia

**Fig. 1:**
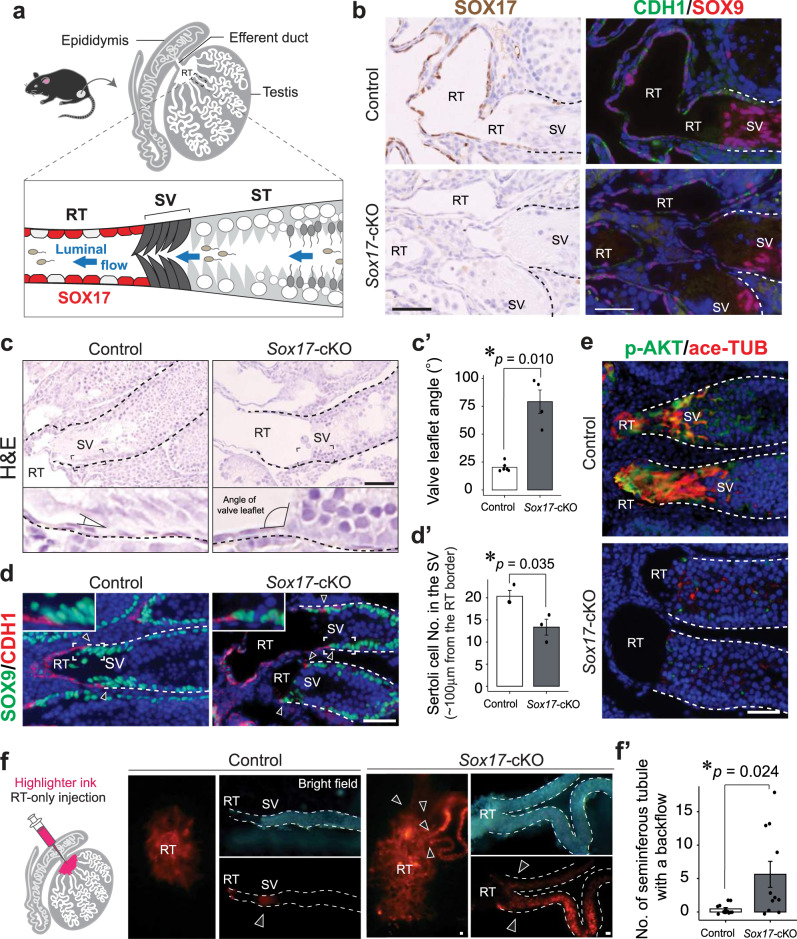
Deformation of the SV in *Sox17-*cKO mice. **a** Schematic illustration of the RT, SV, and ST. **b** SOX17, SOX9 (Sertoli cell & RT marker), and CDH1 (RT marker) immunostaining of two serial sections showing the RT-SV junction at 4 weeks of age. **c** H&E staining showing disrupted geometry of the SV, with an increase in the valve leaflet angle seen in 4-week-old *Sox17-*cKO mice (**c**′). Control *n* = 6. *Sox17-*cKO *n* = 4. **d** SOX9 and CDH1 immunostaining showing reduced Sertoli cell density in the putative SV region in *Sox17-*cKO testis (**d**′). Control *n* = 3. *Sox17-*cKO *n* = 3. **e** SV-specific signals of acetylated tubulin (ace-TUB) and phosphorylated AKT (p-AKT) immunoreactivity were decreased in *Sox17-*cKO mice compared to the controls. **f** Schematic illustration of the injection of dye only into the RT, and surface views of the RT (left) and isolated SV fragments from the testis of which RT was injected with dye (right). The number of labeled STs per testis (i.e., the incidence of fluid backflow from the RT to the ST beyond the SV) increased significantly in *Sox17-*cKO compared to the controls (**f**′). Control *n* = 13. *Sox17-*cKO *n* = 11. Insets show magnified images of the region surrounded by broken rectangles (**c**, **d**). Data are mean ± s.e.m. Comparisons were made using a two-tailed unpaired Student’s *t* test (**c'**, **d'**, **f'**). *P < 0.05. Arrowhead, RT border; broken line, tubular wall; RT, rete testis, SV, Sertoli valve, ST, convoluted seminiferous tubule. “*n*” represents the number of biological replicates. Scale bars, 50 μm.

We have previously shown that the RT, SV, and ST have regionally distinct transcriptome profiles^25^. We identified region-specific upregulation of *Sox17* in the RT (Fig. 1a, b). SOX17 is expressed in RT epithelial cells, as indicated by CDH1 and KRT8 (Fig. 1b, Supplementary Fig. 1a–g) but not in other cells of “gonadosomatic lineage”, such as Sertoli, Leydig, and peritubular myoid cells (Fig. 1b, Supplementary Fig. 1e–g). No *Sox17* signals were observed in the epithelia of the efferent ducts and epididymis, except for the vascular endothelial cells therein^30,31^ (Supplementary Fig. 1a–e). SOX17^+^ RT epithelial cells become evident during E15.5 to 16.5 (Supplementary Fig. 2), leading to the major cell population in the RT after birth that accounts for 70–80% of the total RT epithelial cell population (68.4 ± 2.3%; 79.0 ± 0.8%, 85.4 ± 4.2% at 1, 2, and 4-week old; *n* = 3 each). The proportion of SOX17^+^ RT epithelial cells was similar between germ cell-deficient *W/W*^v^ mutant mice and C57BL/6 wild-type controls (Supplementary Fig. 3a, b), suggesting that *Sox17* expression in the RT is independent of germ cells.

### Defective SV formation in *Sox17*-cKO mice

Given the unique expression of *Sox17* in the RT, we sought to determine whether loss of *Sox17* in the RT affects testicular homeostasis. Since the RT epithelia are the only cell population expressing *Sox17* among the gonadosomatic lineage (denoted “*Nr5a1*”) (Supplementary Fig. 3c)^15–19^, we produced *Nr5a1Cre:Sox17*^*flox/flox*^ (*Sox17-*cKO) mice to deplete *Sox17* in the RT epithelia. Successful deletion of *Sox17* in the RT epithelial cells was demonstrated both in protein (Fig. 1b) and mRNA levels (Supplementary Fig. 3d) in *Sox17-*cKO mice, while no appreciable influence was observed on their expression in other cell types (Supplementary Fig. 3d′). The RT epithelial cells depleted of *Sox17* still showed the expression of RT markers, such as SOX9, CDH1, KRT8, PAX8, and GATA4 (Fig. 1b, Supplementary Fig. 4a) and did not exhibit obvious morphological or proliferative defects in the peripubertal period (Supplementary Fig. 4b–d).

**Fig. 2:**
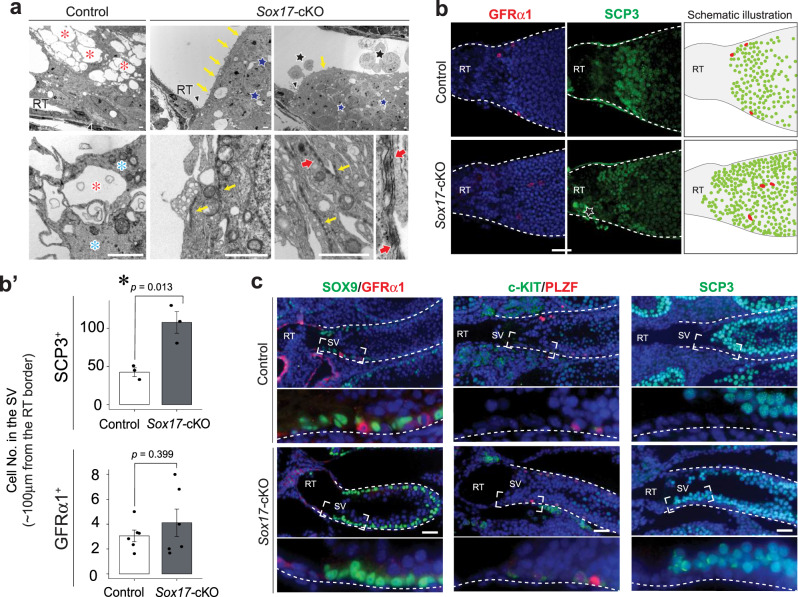
Spermatocytes in the SV of *Sox17-*cKO mice. **a** Transmission electron microscopy (TEM) of Sertoli cells on the border of the RT. Sertoli cells located within the putative SV region in *Sox17-*cKO testes harbored spermatocytes (blue stars) packed into apical cell processes with adherence junction (yellow arrows) constructing ectoplasmic specialization (red arrows), which is not normally observed in SV Sertoli cells. In contrast, SV Sertoli cells in the control did not support spermatocytes, and their cytoplasm was enriched with microtubule bundles (blue asterisk), forming multiple large vacuoles facing the tubular lumen (red asterisk). **b** Whole-mount immunohistochemistry of SCP3 (spermatocyte marker) and GFRα1 (A_undiff_ marker) and schematic illustration of the SV fragments with their quantification (**b**′). Spermatocytes were observed up to the edge of the SV in *Sox17-*cKO mice, while the distribution pattern of A_undiff_ in the putative SV region did not significantly differ between the *Sox17*-cKO and control groups. SCP3: Control *n* = 3, *Sox17-*cKO *n* = 3, GFRα1: Control *n* = 6, *Sox17-*cKO *n* = 6. **c** GFRα1, SOX9, PLZF (A_undiff_ marker), cKIT (A_diff_ marker), and SCP3 immunostaining of three serial testis sections from 4-week-old *Sox17-*cKO mice and their littermate controls. SV epithelia in the control support A_undiff_, whereas the putative SV epithelia in *Sox17-*cKO mice support spermatocytes together with A_diff_. Lower panels in **c** represent magnified images of the region surrounded by broken rectangles in the upper panels. Data are mean ± s.e.m. Comparisons were made using a two-tailed unpaired Student’s *t* test (**b**′). *P < 0.05. Broken line, outlines of the ST; A_undiff_: undifferentiated spermatogonia; A_diff_: differentiating spermatogonia, RT: rete testis, SV: Sertoli valve. “*n*” represents the number of biological replicates. Scale bars, 2 μm (**a**), 50 μm (**b**, **c**).

Considering that the SV region is non-cell-autonomously specified by the RT^25^, we hypothesized that the function of the RT in the SV formation could be impaired in *Sox17-*cKO mice. To test this hypothesis, we examined the phenotype of the SV in 4-week-old *Sox17-*cKO, as the formation of the valve-like structure is completed at this time^25^. Histological analysis revealed that the valve-like structure of the mutant SV was disrupted (Fig. 1c, d). In the normal SV, the valve leaflet is always tilted toward the RT lumen corresponding with the directional luminal fluid flow from the ST to the RT^32^, which is represented as their small angles on the outer tubular surface (less than 30 degrees; Fig. 1c). However, the valve-like structure of the mutant SV was disrupted, in which the angle of the valve leaflet was larger in *Sox17-*cKO than in control animals (Fig. 1c) that implicate the backflow of the fluid from the RT to ST^32^. Concomitantly, the number of Sertoli cells in the putative SV region (~100 μm from the RT) decreased significantly in the mutant mice (Fig. 1d). Few SV-specific ace-TUB and p-AKT signals were detected in the mutant SV, as only a small amount of Sertoli cell cytoplasm protruded towards the RT lumen (Fig. 1e). When the dye was injected into the RT under low pressure, the SV blocked retrograde infusion of the dye thereof into the ST (arrows in Fig. 1f). However, *Sox17-*cKO mice often showed dye backflow from the RT into the ST (Fig. 1f), indicating an impaired function of the SV. Transmission electron microscopy (TEM) confirmed the reduced number of microtubule bundles in the mutant SV Sertoli cells (blue asterisks in Fig. 2a) but also revealed the ectopic formation of actin-based tight junctions (*i.e*., ectoplasmic specialization; red arrows in Fig. 2a), which is not normally observed in modified Sertoli cells in the SV^26,33–35^. Notably, spermatocytes were ectopically observed in the adluminal compartment of the mutant SV (Fig. 2a–c), suggesting that the disrupted microenvironment of the *Sox17-*cKO SV permits the differentiation of its local spermatogonia^25^. Taken together, these data suggest that *Sox17*^+^ RT epithelia contribute to the proper formation of the SV, presumably in a paracrine manner.

### Comprehensive molecular characterization of the *Sox17-*cKO mouse testis by scRNA-seq

**Fig. 3:**
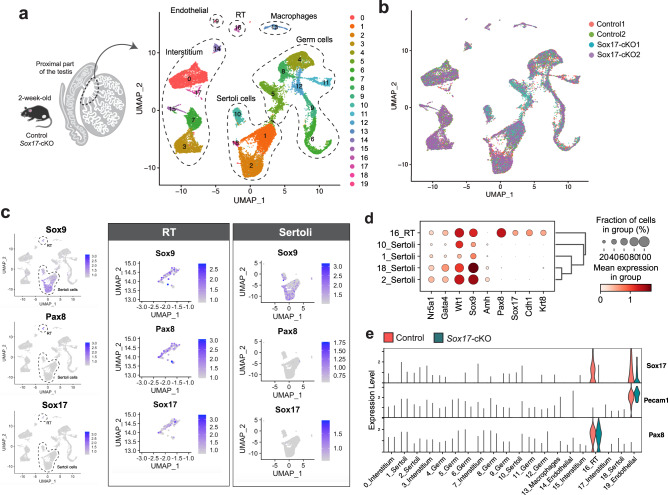
Molecular characterization of the testis of *Sox17-*cKO mice by scRNA-seq. **a**, **b** Schematic illustration of the experiment and Uniform Manifold Approximation and Projection (UMAP) plot of cells isolated from the proximal part of the testis of P14 *Sox17-*cKO and controls, showing twenty clusters (**a**) and the distribution of cells from each sample (**b**). Control *n* = 2, *Sox17-*cKO *n* = 2. **c** UMAP plot showing the expression level of *Sox9, Pax8, and Sox17* in the control samples. The right two panels show the expression of each gene in the extracted clusters of the RT (cluster 16) and Sertoli cells (clusters 1, 2, 10, 18). **d** Dot plots representing the expression levels of marker genes for Sertoli cells (*Amh*) and RT epithelial cells (*Krt8, Pax8*) together with their common marker genes (*Nr5a1, Gata4, Wt1, Sox9*) in the clusters expressing *Sox9* shown in (**c**) (clusters 1, 2, 10, 16, 18). **e** A violin plot showing the expression level of *Sox17* in each cluster, together with *Pecam1* (vascular endothelial cell marker) and *Pax8* (RT marker). “*n*” represents the number of biological replicates.

To check for initial defects in the RT and SVs of *Sox17-*cKO testes, we performed single-cell RNA sequencing (scRNA-seq) on the proximal part of the testes collected from the control and cKO testes at P14 (Fig. 3a). After filtering out low-quality cells and doublets, 22,167 cells were retained for subsequent analysis. First, we employed a nonlinear dimensionality-reduction technique called Uniform Manifold Approximation and Projection (UMAP) to visualize the data^36^. As a result, we identified cell clusters corresponding to twenty distinct cell types (Fig. 3a, Supplementary Fig. 5). Visualization of the sample origin in the UMAP plot showed subtle alteration in their cell distribution in each sample (Fig. 3b, Supplementary Fig. 5a, b). To identify cell clusters corresponding to the RT epithelial cells and Sertoli cells, we next performed a differentially expressed gene (DEG) analysis to examine enriched gene expression in each cluster (Supplementary Data 1). As a result, we identified the distinct expression of *Sox9* in 1, 2, 10, 16, and 18 (Fig. 3c, d, Supplementary Fig. 5c–e). Among them, cluster 16 was distinguished by the high expression of both *Pax8, Cdh1, Krt8*, and *Sox17* (Fig. 3c–e, Supplementary Fig. 5c), while *Sox17* expression was specifically decreased in cluster 16 (*Pecam1*^*−*^*/Pax8*^+^) of *Sox17-cKO* samples (Fig. 3e). These data suggest that cluster 16 defines the RT epithelial cells, while clusters 1, 2, 10, and 18 represent Sertoli cells, as indicated by their distinct transcriptome profiles (Supplementary Fig. 5c, d). Aside from clusters 1, 2, 10, 16, and 18, we identified clusters 4–6, 8, 9, 11, 12 as germ cells, clusters 0, 3, 7, 15, and 17 as testicular interstitial cells, cluster 19 as endothelial cells, and cluster 13 as macrophages by using the representative marker genes for each cell types as a reference (Supplementary Fig. 5e, f).

**Fig. 4:**
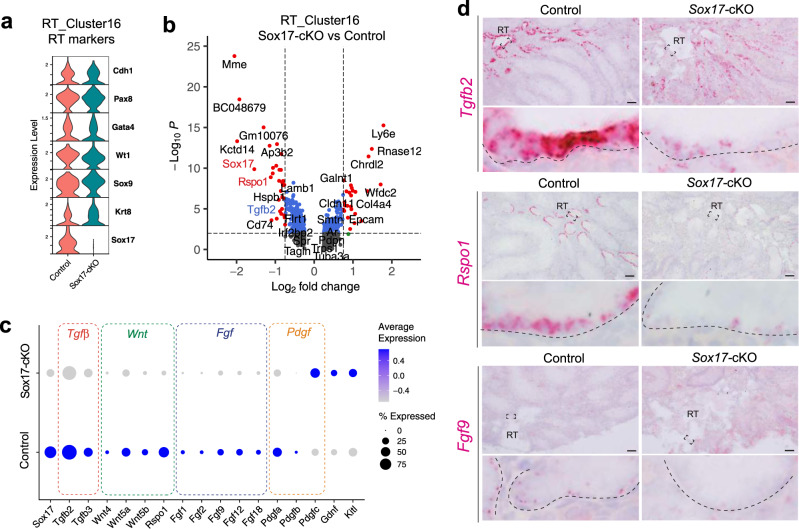
Decreased expression of various growth factor genes in the RT of *Sox17-*cKO mice. **a** Violin plots showing the conserved marker gene expression of the RT epithelial cells (cluster 16) of *Sox17-*cKO mice. **b** A volcano plot illustrating the DEGs of the RT between *Sox17-*cKO mice and the controls. **c** A dot plot representing the expression profiles of genes related to paracrine signaling in cluster 16. The expression level of several genes encoding a paracrine factor was relatively low in the RT of *Sox17-*cKO mice compared to the controls. **d** in situ hybridization of *Tgfb2, Rspo1*, and *Fgf9* in the RT of 4-week-old *Sox17-*cKO mice and the controls, confirming their reduced expression in the RT of *Sox17-*cKO mice. Lower panels in (**d)** represent magnified images of the region surrounded by broken rectangles in the upper panels. Broken line, outlines of the RT; RT, rete tests. Scale bars, 50 μm (**d**).

To explore the initial effect of *Sox17* depletion, we next focused on cluster 16 that represents RT epithelial cells. Being consistent with the histological observations, the RT epithelial cells of *Sox17*-cKO mice conserved their marker gene expression (Fig. 4a). On the other hand, DEG analysis identified reduced expression levels of several genes encoding paracrine factors such as *transforming growth factor-beta 2* (*Tgfb2), R-spondin 1(Rspo1)*, and *platelet-derived growth factor subunit a* (*Pdgfa)* in the RT of *Sox17-*cKO mice (Fig. 4b, c. Supplementary Data 2). Several other growth factors including *Fgf* and *Wnt* genes, such as *Fgf2*, *Fgf9*, *Fgf12*, *Wnt4*, and *Wnt5a*, also show a tendency to decrease their expression levels in *Sox17-*cKO mice (Fig. 4c), albeit with a slightly higher expression of some growth factors such as *Gdnf* and *Kitl* (Fig. 4c). In situ hybridization also confirmed reduced signals of *Tgfb2*, *Rspo1*, and *Fgf9* in the RT epithelia of *Sox17-*cKO mice compared to those in the controls (Fig. 4d). These data demonstrate the decreased growth factor production in the RT of *Sox17*-cKO mice, that can impact the cells located adjacent to them: SV Sertoli cells.

**Fig. 5:**
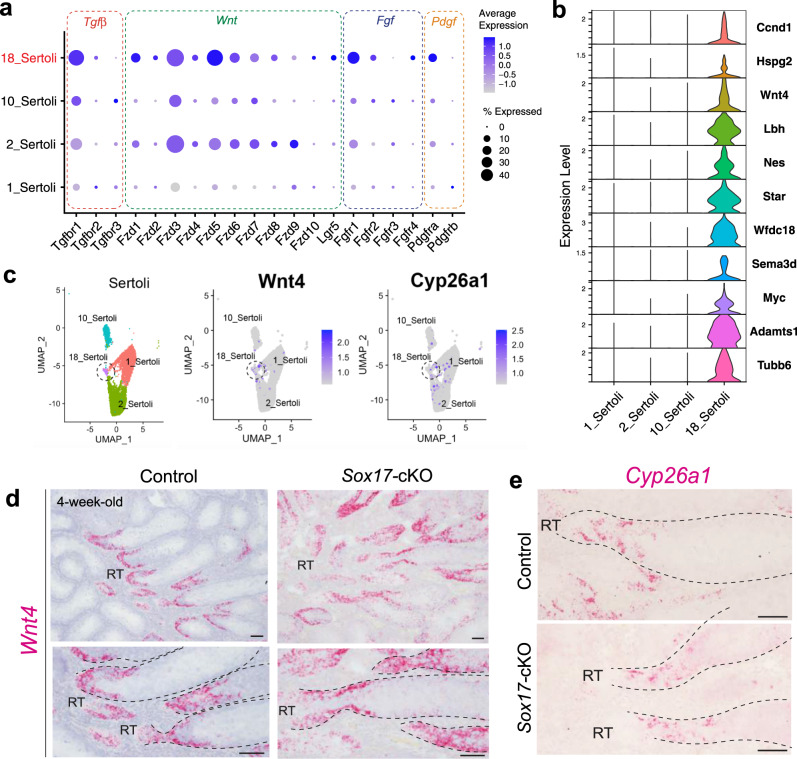
Characterization of the SV Sertoli cells in the scRNA-seq data. **a** A dot plot representing the expression profiles of genes related to the reception of growth factors shown in Fig. 4c, in Sertoli cell clusters. Cluster 18 showed relatively high expression of these receptor genes. **b** Expression of selected marker genes of cluster 18 represented as violin plots. **c** UMAP plots showing the distribution of *Wnt4* (cluster 18 maker) and *Cyp26a1* (SV marker in adult) expressing cells in Sertoli cell clusters. **d**, **e** In situ hybridization of *Wnt4*, and *Cyp26a1* in the presumptive SV region of 4-week-old *Sox17-*cKO mice and the controls. *Wnt4* expression was regionally restricted at the terminal end of the ST. Sertoli cells expressing *Wnt4* and *Cyp26a1* were both present in the SV region even in *Sox17-*cKO mice, albeit with the deformation of their valve-like structure. The lower panels in (**d**) represent high magnification images of the region surrounded by broken rectangles in the upper panels. Broken line, outlines of the ST; RT: rete testis, SV: Sertoli valve. Scale bars, 50 μm.

Noting the decreased expression of genes encoding growth factors at the RT of *Sox17*-cKO mice (*i.e*., *Tgfb, Wnt, Fgf*, and *Pdgf*), we next sought to determine which Sertoli cell population expresses the cognate receptors to receive these ligands. Interestingly, Sertoli cells that constitute cluster 18 tend to have higher expression levels of genes encoding the receptors that correspond to the growth factors secreted from the RT (Fig. 5a). Among them, we appreciated the high expression of *Fgfr1* in cluster 18, which is reported to be highly expressed in the SV Sertoli cells^25^, DEG analysis of cluster 18 from other Sertoli cell clusters resulted in the identification of its unique marker genes (Fig. 5b, Supplementary Data 3), among which *Cyclin D1* (*Ccnd1)* has been reported to be a marker for the SV Sertoli cells^24^. In addition, *Hspg2* contributes to the heparan sulfate proteoglycan (HSPG) that modulates the activity of multiple diffusible factors (*e.g*., FGFs) which is enriched at the basement membrane outlining the SV^24,25^. These data suggest that cluster 18 represents Sertoli cells that comprise the SV. Consistently, UMAP representation showed that the cells expressing *Wnt4* (a representative marker gene for cluster 18, Fig. 5b) and *Cyp26a1* (a gene expressed in adult SV cells^25^) are distributed around cluster 18 (Fig. 5c). We further demonstrated the region-specific expression of *Wnt4* at the SV by in situ hybridization at 4-week-old mice (Fig. 5d), as well as *Cyp26a1* (Fig. 5e). As a result, Sertoli cells which express *Wnt4* and *Cyp26a1* were present even in the absence of *Sox17* at the RT (Fig. 5d, e). There are few DEGs between *Sox17-*cKO and controls in cluster 18, as well as the other three clusters at P14 (Supplementary Data 4). These data suggest that the initial specification of the SV Sertoli cells takes place regardless of *Sox17*^+^ RT, yet its absence disrupts the formation/maintenance of the valve-like structure at the SV by 4-week-old.

### Deformation of the SV impairs spermiation

**Fig. 6:**
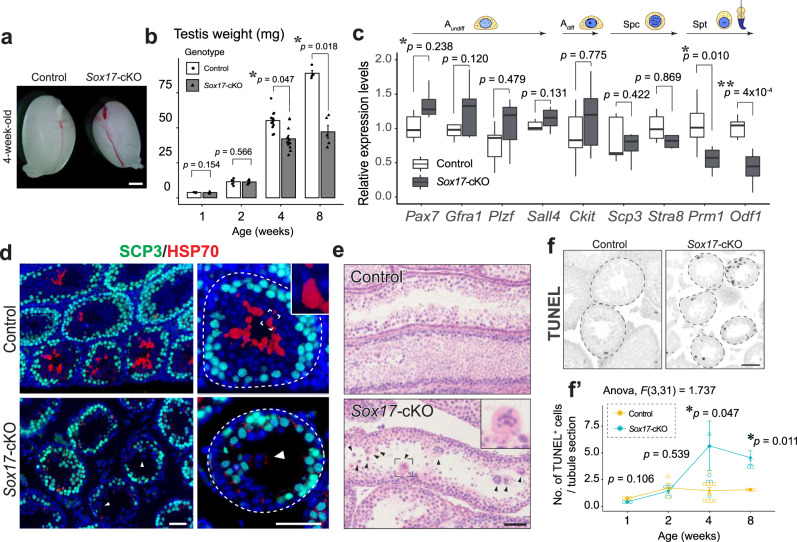
Aberrant spermiogenesis in *Sox17-*cKO testes. **a** Gross morphology of the testis from 4-week-old *Sox17-*cKO and their littermate controls. **b** The decrease of testicular weight in *Sox17-*cKO mice from 4 weeks of age onward. 1wk: Control *n* = 3, *Sox17-*cKO *n* = 4, 2wk: Control *n* = 5, *Sox17-*cKO *n* = 4, 4wk: Control *n* = 12, *Sox17-*cKO *n* = 11, 8wk: Control *n* = 4, *Sox17-*cKO *n* = 5. **c** RT-qPCR analysis of the whole testis from 4-week-old *Sox17-*cKO and control group animals. Expression levels of spermatid-specific marker genes were significantly decreased in *Sox17-*cKO mice, among the marker genes for germ cells at each developmental stage, as shown at the top of the figure. Control *n* = 6, *Sox17-*cKO *n* = 6. **d**, **e** SCP3 and HSP70 immunostaining (**d**) and H&E staining (**e**) of testicular sections from 4-week-old *Sox17-*cKO mice and controls. Few elongated spermatids were marked by HSP70 during the first wave of spermatogenesis in the *Sox17-*cKO testes (**d**). Immature spermatids often detached from the ST lumen to form multinucleated syncytia (arrowheads in (**d**) and (**e**)). **f** A significant increase in TUNEL^+^ apoptotic cells in the STs of *Sox17*-cKO testes was seen from 4 weeks of age onward (**f**′). 1wk: Control *n* = 3, *Sox17-*cKO *n* = 3, 2wk: Control *n* = 5, *Sox17-*cKO *n* = 4, 4wk: Control *n* = 7, *Sox17-*cKO *n* = 10, 8wk: Control *n* = 4, *Sox17-*cKO *n* = 3. Insets show magnified images of the region surrounded by broken rectangles (**d**, **e**). A_undiff_, undifferentiated spermatogonia; A_diff_, differentiating spermatogonia; Spc, spermatocyte; Spt, spermatids. Broken line, tubular wall. Data are mean ± s.e.m. (**b**, **f**) or as box-and-whisker plots displaying median, interquartile range (boxes), and minima and maxima (whiskers) (**c**). Comparisons were made using a two-tailed unpaired Student’s *t* test (**b**, **c)** or two-way repeated measures ANOVA with Bonferroni’s two-sided multiple comparisons (**f**′). “*n*” represents the number of biological replicates. *P < 0.05. Scale bars, 1 mm (**a**), 50 μm (**d**–**f**).

To elucidate the physiological function of the RT and SV in the testis, we examined the effect of *Sox17-*cKO on spermatogenesis in the ST (Fig. 6, Supplementary Fig. 6). Surprisingly, *Sox17-*cKO mice were completely infertile. Hardly any sperm, albeit with some immature germ cells, were observed in the epididymis at 8 weeks of age (Supplementary Fig. 6a, b). Although no morphological abnormalities were apparent in the prepubertal mutant mouse testis at 1 and 2 weeks of age (Supplementary Fig. 6c–f), the testis of 4-week-old *Sox17-*cKO mice were smaller compared to their wild-type littermates (Fig. 6a, b, Supplementary Fig. 6c), coinciding with the emergence of elongated spermatids during the first wave of spermatogenesis^37^. Gene expression levels of spermatid markers (*i.e., Odf1* and *Prm1*) decreased significantly in *Sox17-*cKO mice at 4 weeks of age (Fig. 6c), and fewer HSP70^+^ spermatids were observed in a testis section of 4-week-old *Sox17-*cKO mice (Fig. 6d). Notably, STs of *Sox17-*cKO mice often had multinucleated spermatid syncytia (Fig. 6e), which are commonly observed in mice with spermiogenesis or spermiation defects^13,38^. The number of TUNEL^+^ apoptotic spermatogenic cells increased significantly in *Sox17*-cKO mice from 4 weeks of age onwards (Fig. 6f), which potentially reflected increased cell death due to spermiogenesis defects. In the drastic phenotype, Sertoli cells that supported the spermatids did not show hardly any change in their distribution pattern and marker gene expressions (Supplementary Fig. 6g), together with intact blood-testis-barrier (BTB) visualized by tracer molecule biotin (Supplementary Fig. 6h). These findings suggest that the abnormal release of immature round spermatids led to male infertility, as a consequence of the disrupted testicular fluid dynamics caused by the valve deformity in *Sox17*-cKO mice.

**Fig. 7:**
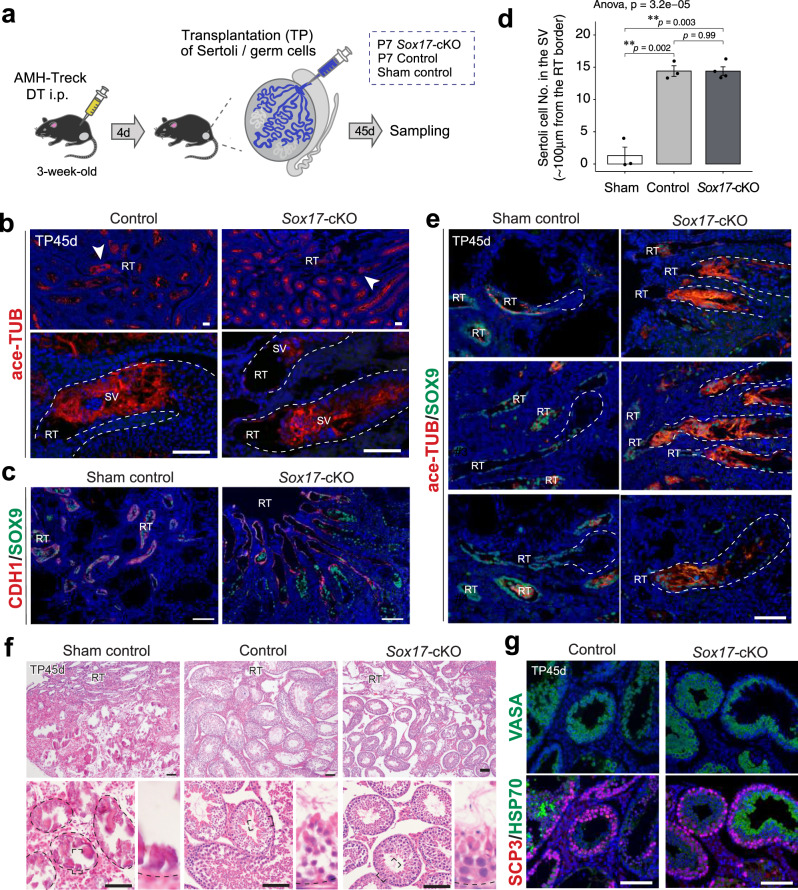
Reconstruction of the SV and spermatogenesis by the Sertoli cells of *Sox17-*cKO mice. **a** A schematic of Sertoli cell ablation/transplantation experiment. Sertoli and germ cells of *Sox17-*cKO mice, their littermate wild-type control (control), or trypan blue (sham control) were injected into the testis of *AMH-Treck* mice, respectively. In this experiment, *AMH-Treck* males were pretreated with diphtheria toxin (DT) to ablate the endogenous Sertoli cells on 4 days prior to the transplantation. **b**–**e** Sertoli cells of *Sox17*-cKO mice reconstituted ace-TUB^+^ valve-like structure at the presumptive SV region adjacent to the host RT (SOX9^+^/CDH1^+^). Sertoli cells of control mice also constructed the SV (b), while sham control testis did not show any valve-like structure at the SV (**c**, **e**). The number of Sertoli cells in the putative SV region was similar between the *Sox17-*cKO group and the control group (**d**). Sham control *n* = 3. Control *n* = 3. *Sox17-*cKO *n* = 4. In (**b**), (**c**), and (**e**), the testes shown in the left panel and the right panel are contralateral testis from the same animal. In e, the top, middle, and bottom panels show data obtained from different mice as a biological replicate. **f** Restored spermatogenesis in *AMH-Treck* mouse testes transplanted with Sertoli cells derived from *Sox17-*cKO mice. Spermatozoa were observed in the ST epithelium of the testis transplanted with Sertoli cells derived from the control and *Sox17-*cKO mice. No spermatogenic activity was observed in the ST of sham controls, where most of the ST underwent calcification. **g** Immunohistochemistry of VASA, SCP3, and HSP70 in the testis section of *AMH-Treck* mice. Note the presence of elongated spermatids strongly marked by HSP70. Lower panels in (**b**) show the magnified image of the region indicated by arrowheads in the upper panels. The lower panels in f show the testis section at high magnification, respectively. The upper and lower panels in **g** show images of serials sections. Data are mean ± s.e.m. Broken line, outlines of the ST. RT rete tests, SV Sertoli valve, ST seminiferous tubule. TP, post-transplantation. “*n*” represents the number of biological replicates. Scale bars, 100 μm.

Finally, we sought to determine whether Sertoli and germ cells of *Sox17-*cKO mice have the capability to undergo proper SV formation and spermiogenesis when they are transplanted into the testis with normal *Sox17*^+^ RT. To address this question, we used hAMH promoter-driven toxin receptor-mediated cell knockout (*AMH-Treck*) mice, in which endogenous Sertoli cells (GFP^+^) can be ablated after administering diphtheria toxin (DT)^39,40^. In brief, we prepared Sertoli and germ cell suspensions from the ST of *Sox17*-cKO testes and then transplanted them into the empty ST of DT-pretreated *AMH-Treck* host testes without their endogenous Sertoli cells^25,39^ (Fig. 7a). At 45 days post-transplantation, the *AMH-Treck* mouse testis transplanted with the cell suspension, collected either from *Sox17*-cKO mice or their littermate controls, successfully reconstructed ace-TUB^+^ valve-like structures with Sertoli cells at the presumptive SV region next to the RT (Fig. 7b–e). In contrast, the DT-treated *AMH-Treck* mouse testes without any cell transplantation (sham control) lacked either the SV or spermatogenesis in the ST, which resulted in the calcification of most parts of the ST (Fig. 7c–f) albeit with a few GFP^+^ Sertoli cells remained in their seminiferous epithelia (Supplementary Fig. 7). The *Sox17-*cKO Sertoli and germ cells transplanted into *AMH-Treck* testes further restored spermiogenesis in the ST with a reconstituted SV (Fig. 7f, g). The STs with restored spermatogenesis contained hardly any GFP^+^ cells (Supplementary Fig. 7), which confirms that the recovered spermatogenesis in the testes transplanted is supported by GFP^−^ donor-derived Sertoli cells. These findings demonstrate that the *Sox17*^+^ RT induces the proper formation of SV and supports complete spermiogenesis in the ST.

## Discussion

**Fig. 8:**
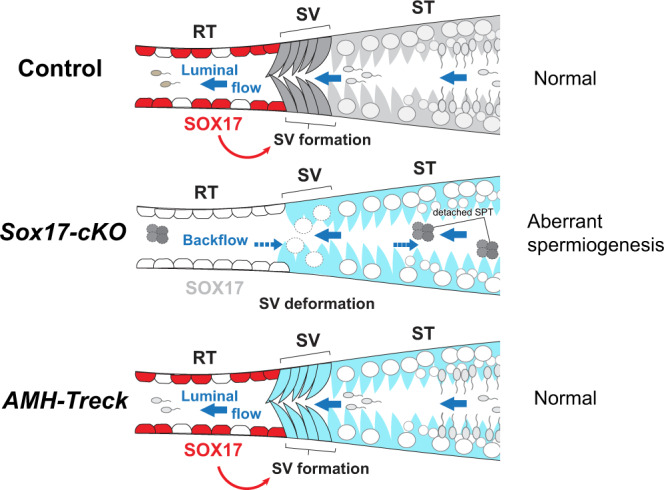
SOX17 acts as a positive regulator of the formation of the SV. Schematic diagram summarizing the findings of this study. *Sox17* expression in the RT modulates the formation of the SV, which regulates the unidirectional outflow of the luminal fluid from the ST into the RT. Ablation of *Sox17* in the RT results in deformation of the SV and backflow of the luminal fluid from the RT to the ST. This causes aberrant spermiogenesis in the STs. Relocation of Sertoli cells of *Sox17-*cKO mice into Sertoli cell-ablated *AMH-Treck* mouse testis results in the reconstitution of the SV, while restoring spermatogenesis in the ST. Red arrows indicate putative paracrine factors downstream of *Sox17* action that promote SV formation. RT rete testis, ST convoluted seminiferous tubule, SV Sertoli valve.

The RT and SV structures in the testis are evolutionally conserved among various mammalian species including humans^26,34,35^; however, their significance in male gametogenesis has been largely overlooked for more than half a century. Here, we demonstrated the key role of the *Sox17*^+^ RT epithelia in SV valve formation and subsequent healthy spermiogenesis in the ST (Fig. 8). The main function of a valve is to orient fluid flow in a single direction by physically preventing backflow in the channel, hence its deformation leads to regurgitation in various organs^21,22^. Likewise, deformation of the valve-like structure at the SV of *Sox17*-cKO testes permits the backflow of luminal fluid from the RT to the ST (Fig. 1f). This backflow makes the ST epithelium exposed to aberrant fluid dynamics/composition, which can contribute to the abnormal release of immature spermatids into the lumen of the ST (Fig. 6d, e). The physical force of repeatedly reversed luminal fluid flow has been reported to force spermiation in the STs^41^, while shear forces of fast fluid flow in the STs induce detachment of germ cells from the seminiferous epithelium^41^. These observations suggest that altered sheer stress caused by the disrupted fluid dynamics in the ST can be responsible for the spermiation defects observed in *Sox17-*cKO mice. In addition, the compositions of luminal fluid in the RT and the ST differ^42,43^, and the cyclical distribution of various factors in the STs is tightly regulated in a seminiferous epithelial cycle-dependent manner^9,44^. Such a regionally distinct fluid composition/microenvironment in the testis could also be disturbed by SV disruption in *Sox17-*cKO mice. Of note, SCP3^+^ cells ectopically observed in the SV of *Sox17-*cKO mice (Fig. 2) could reflect the altered microenvironment around the SV, caused either by fluid backflow or via altered characteristics of the Sertoli cells comprising the SV. Because the increase of RA levels in the SV is sufficient for the emergence of SCP3^+^ cells at the SV^25^, it nevertheless suggests that the RA levels at the SV of *Sox17-*cKO mice are sufficient to allow the differentiation of local spermatogonia.

A rescue experiment with *AMH-Treck* mice demonstrated that Sertoli cells of *Sox17-*cKO mice could form a functional SV when transplanted next to the *Sox17*^*+*^ RT (Fig. 7). This suggests that *Sox17*^+^ RT epithelia are responsible for the non-cell-autonomous formation of the SV. Previous studies suggested that SV formation is orchestrated by several growth factors and chemokines such as FGFs, RA, and WNT^18,25^. Here, our scRNA-seq analysis revealed that *Sox17*-null RT had reduced expression of several growth factor genes such as *Tgfb2, Rspo1*, and *Pdgfa*, together with decreasing tendencies in *Fgf* and *Wnt* family ligands. *Tgfb2* and *Pdgfa* are known to be involved in valve formation of the heat and aorta^45,46^, while they can contribute to the activation of PI3K-AKT signaling^47,48^ which is characteristic of the SV Sertoli cells. Similarly, FGF9 activates AKT in Sertoli cells^25^. Therefore, reduced expression of *Tgfb2*, *Pdgfa*, and *Fgfs* in the RT might contribute to the reduced AKT phosphorylation in the SV Sertoli cells (Fig. 1e). This suggests that several growth factors secreted from the RT epithelial cells must collaborate to orchestrate the formation of the SV, which can be impaired by *Sox17* deficiency in the RT epithelia.

This study revealed distinct expression of *Rspo1* (an enhancer of WNT signaling^49,50^) at the RT and *Wnt4* expression in the SV Sertoli cells, respectively (Fig. 4d, 5d). Together with SV region-specific enrichment of HSPG^24,25^, which serves as a co-receptor for RSPO^50^, this implies unique WNT signaling states at the RT–SV interface in the postnatal mouse testis. Interestingly, constitutive activation of WNT/β-catenin signals in postnatal Sertoli cells conserves their proliferative activity and high GDNF expression, while compromising their differentiation into ST Sertoli cells even after sexual maturation^51^. This is consistent with the reported characteristics of the SV Sertoli cells^24–26^, thus implicating the roles of WNT signaling in SV formation and/or maintenance. In the RT of *Sox17-*cKO mice, the expression levels of *Rspo1* were significantly decreased together with several *Wnt* ligand genes (Fig. 4b–d). This suggests that the region-specific WNT signaling states at the SV could be impaired by the deficiency of RT-derived RSPO1, which could be associated with the defective formation of a valve-like structure in *Sox17-*cKO mice. On the other hand, in embryonic gonads, the supporting-like cells show transiently high expression of *Wnt4* around E11.5 that contributes to the initial formation of RT at E14.5^18^ whereas *Sox17* starts to be expressed in the RT from E15.5 (Supplementary Fig. 2). This implies a distinct function of WNT signaling between the embryonic gonad and postnatal testes in mice.

In *Sox17*-cKO mice, deletion of *Sox17* in the RT epithelia caused little alteration in their ultrastructural structure and their marker gene expressions (Fig. 4a and Supplementary Fig. 4), but also exerted no appreciable defects in the excurrent duct function to transport the spermatogenic cells (Supplementary Fig. 6b). Because the dye we injected into the RT did not leak into the testicular interstitium, the barrier function of the RT appeared to remain intact (Fig. 1f). These data suggest that the mutant mice could develop RT epithelia regardless of *Sox17* expression. Such defects in *Sox17*-null RT could reflect a genetic redundancy within SOX family members in the RT epithelia. The RT expresses *Sox9* (Fig. 1b) and *Sox3* (Supplementary Data 1) in addition to *Sox17*. Indeed, previous studies have shown the compensational roles among Sox family members. *Sox9* and *Sox8* are co-expressed in Sertoli cells, where these two genes have redundant functions in the maintenance of the tubular structures of the STs^52^. *Sox17* is expressed in the luminal epithelial cells of uterine tubules, and the heterozygous deletion of *Sox17* leads to the upregulation of SOX9 therein in a compensational manner^53^. Such compensational roles of SOX-encoding genes likely assure the structural integrity of the RT, even in the absence of *Sox17*.

In conclusion, we have demonstrated the expression of *Sox17* in the RT and revealed its crucial role in SV formation and spermiogenesis in STs. Deletion of *Sox17* in the RT disrupted the specification of modified Sertoli cells that form the SV, which caused a backflow of RT fluid; this led to aberrant detachment of immature spermatids in the STs (Fig. 8). Conversely, relocation of Sertoli cells from *Sox17-*cKO mice next to *Sox17*^+^ RT epithelial cells promoted reconstruction of the SV, and further enabled healthy spermatogenesis in the ST (Fig. 8). This study shows the importance of the RT in SV formation and elucidated the physiological function of the SV in preventing the backflow of seminiferous tubular fluid, which impacts spermiogenesis in the entire organ. It might be possible to exploit the ability of the RT and/or SV to modulate testicular fluid dynamics to improve spermatogenesis in vitro.

## Methods

### Animals

*Nr5a1Cre*^53,54^ and *Sox17*^*flox/flox*^ ^55^ mice were obtained from the Jackson Laboratory. *Nr5a1:Sox17*^*flox/flox*^ (*Sox17-*cKO) and *Sox17*^*flox/flox*^ (control) mice were generated by crossing a *Sox17*^*flox/flox*^ male and *Nr5a1Cre:Sox17*^*flox/flox*^ female. *Sox17*-eGFP knock-in mice^56^ were used to visualize *Sox17* expression in the testis (Supplementary Fig. 1a–f). *Nr5a1Cre:ROSA26*^*tdTomato*^ mice were used to visualize *Nr5a1* expression in the RT (Supplementary Fig. 3c). *WBB6F1/Kit-KitW/KitW*^*v*^*/Slc* (*W/W*^*v*^, SLC) mice were used as a germ cell-less mutant mice (Supplementary Fig. 3a, b). To examine the initial phenotype of the mutant mice and exclude secondary effects of spermatozoa, detached germ cells, and their cell debris (particularly in *Sox17-*cKO mice), we analyzed the mutant mice at 4 weeks of age, when the valve-like structure at the SV was developing^25^. Animals were provided with water and commercial laboratory mouse chow ad libitum and were housed under controlled lighting conditions (daily light from 08:00 to 20:00) with temperatures of 18–23 °C and 40–60% humidity. All animal experiments were performed following the Guidelines for Animal Use and Experimentation at the University of Tokyo, and all experimental procedures performed herein were approved by the Institutional Animal Care and Use Committee, Graduate School of Agricultural and Life Sciences, University of Tokyo (approval IDs: P13-762 and P13-764).

### Histology and immunohistochemistry

Testes were fixed in 4% paraformaldehyde (PFA) at 4 °C overnight, dehydrated through an ethanol series, and embedded in paraffin. The paraffin sections (4 μm thickness) were subjected to hematoxylin and eosin (H&E) staining and immunohistochemistry. The sections were incubated overnight at 4 °C with the following primary antibodies [format: host anti-protein (company, catalog number, dilution)]: mouse monoclonal anti-ace-TUB (Sigma, T6793, 1:200), mouse monoclonal anti-αSMA/ACTA2 (Sigma, A5228, 1:500), goat polyclonal anti-AMH (Santa Cruz, sc6886, 1:200), mouse monoclonal anti-CDH1 (BD Transduction lab, 610181, 1:400), goad polyclonal anti-c-KIT (R&D systems, AF1356, 1:200), rabbit monoclonal anti-EPCAM (Abcam, ab32392, 1:100), goat polyclonal anti-GATA4 (Santa Cruz, sc1237, 1:200), mouse monoclonal anti-GATA4 (Santa Cruz, sc25310, 1:100), rabbit polyclonal anti-GFP (MBL, 598, 1:200), goat polyclonal GFRα1 (R&D systems, AF560, 1:100), rabbit polyclonal anti-HSP70 (Abcam, ab79852, 1:1000), rabbit polyclonal anti-KI67 (Abcam, ab15580, 1:400), rabbit monoclonal anti-KRT8 (Abcam, ab53280, 1:200), rabbit polyclonal LAMININ (Abcam, ab11575, 1:200), rabbit monoclonal anti-p-AKT (Cell Signaling, 4060, 1:100), mouse monoclonal anti-PAX8 (Abcam, ab53490, 1:100), rat monoclonal PECAM1 (Invitrogen, 14-0311-82, 1:100), rabbit polyclonal anti-PLZF (Santa Cruz, sc22839, 1:200), mouse monoclonal SCP3 (Santa Cruz, sc74569, 1:500), goat polyclonal anti-SOX17 (R&D systems, AF1924, 1:200), rabbit polyclonal anti-SOX9 (Millipore, AB5535, 1:400), rabbit polyclonal anti-STAR (Cell Signaling, 8449, 1:100), rabbit polyclonal anti-VASA/MVH (Abcam, ab13840, 1:10000). The AlexaFluor 488 Tyramide Signal Amplification kit (Invitrogen) was used to visualize the immunostaining reaction of the anti-p-AKT primary antibody. TUNEL assay was performed using the DeadEnd™ Fluorometric TUNEL System (Promega) according to the manufacturer’s instructions. For whole-mount analysis, testes were fixed in 4% PFA at 4 °C overnight and the tunica albuginea was removed and dispersed in cold PBS. The ST fragments were isolated manually, permeabilized with a gradient methanol series, and incubated overnight at 4 °C with each of the primary antibodies (1:100 dilution). Immunoreactions were visualized using Alexa fluor-conjugated secondary antibodies at 1:200 dilution as follows; chicken anti-mouse Alexa 488 conjugated (Invitrogen, A-21200), chicken anti-rabbit Alexa 488 conjugated (Invitrogen, A-21441), donkey anti-goat Alexa 488 conjugated (Invitrogen, A-11058), goat anti-mouse Alexa 594 conjugated (Invitrogen, A-11032), chicken anti-rabbit Alexa 594 conjugate (Invitrogen, A-21442), donkey anti-goat Alexa 594 conjugate (Invitrogen, A-11058), donkey anti-rabbit Alexa 680 (Invitrogen, A-10043) or biotin-conjugated secondary antibodies at 1:200 dilution as follows: horse anti-mouse IgG biotinylated (Vector Laboratories, BA-2001), goat anti-rabbit IgG biotinylated (Vector Laboratories, BA-1000), rabbit anti-goat IgG biotinylated (Vector Laboratories, BA-5000). The reaction of biotin-conjugated secondary antibodies was visualized with the Elite ABC Kit (Vector Laboratories). Samples were analyzed by fluorescence microscopy (BX51N-34-FL-2) or Leica TCS SP8 confocal laser microscopy. Testes from at least four animals in each age group were examined, and tissue sections from *Sox17-*cKO and their littermate controls were processed in parallel to ensure the reproducibility of the results.

### Electron microscopy

Testes were trimmed and fixed in 2.5% glutaraldehyde in 0.1 M sodium cacodylate buffer (pH 7.4) overnight, post-fixed with 0.5% OsO4 suspended in 0.1 M phosphate buffer (pH 7.3) for 30 min and dehydrated through a graded ethanol series. After passing through propylene oxide, the tissues were embedded in Epon 812. Ultrathin sections were cut, stained with uranyl acetate and lead citrate, and observed by TEM (JEM-1010; JEOL).

### RNA in situ hybridization

Testes were fixed in 10% neutral buffered formalin at room temperature overnight, dehydrated through an ethanol series, and embedded in paraffin. The paraffin sections (4 μm thickness) were processed for RNA in situ detection using the RNAscope 2.5HDAssay Red Manual (ACDBio) according to the manufacturer’s instructions. The probes used in this study are listed in Supplementary Table 1.

### RT-qPCR

Total RNA was extracted from the whole testis using the RNeasy Mini Kit (Qiagen), followed by reverse transcription using the High-Capacity cDNA synthesis kit (Thermo Fisher Scientific). RT-qPCR was performed using ABI 7500 Fast PCR (ABI) in the SYBR Green system and the primers listed in Supplementary Table 2. The expression levels of all genes are presented relative to *Actb1* expression.

### Dye injection into the RT

Water-soluble red highlighter ink (Mitsubishi) was used to label the fluid^57^. The ink was taken from a highlighter pen, diluted 1:2 in 0.4% Trypan blue solution (Gibco) in PBS, and injected via the efferent duct using a microcapillary under a dissecting microscope^25,39^. In this experiment, approximately 1 μl of dye solution was injected into the RT to label the fluid (Fig. 1f). Soon after the injection, the animals were euthanized, and the testes were collected and fixed in 4% PFA at room temperature for 30 min. The testes were rinsed three times in cold PBS and the tunica albuginea was then removed to visualize the area labeled with dye (Fig. 1f). All the labeled STs (i.e., the SVs that failed to prevent fluid backflow from the RT) were counted (*n* = 13 and *n* = 11 testis in control and *Sox17-*cKO mice, respectively).

### Testis sample preparation for scRNA-seq

The samples analyzed by scRNA-seq consisted of testicular cells pooled from three pups per sample. Testes from 2-week-old (P14) *Sox17-*cKO and control mice were collected, and the region containing the RT and adjacent ST was manually dissected (Fig. 3a), rinsed extensively in Hank’s buffered salt solution (HBSS), digested at 36 °C with a mixture of collagenase IV (2 mg/ml) and hydrogenase (2 mg/ml) for 30 min, and centrifuged at 300 × *g* for 1 min. The supernatant containing the interstitial cell population was discarded. The cells were digested in 0.125% Trypsin/EDTA (Gibco) for 5 min, and the reaction was inhibited with 10% fetal bovine serum (FBS). DNase I was added at all digestion steps (1 mg/ml). The cells were washed in cold HBSS, filtered through a 40-mm cell strainer (Flowmi), re-suspended in 0.1% BSA/HBSS, and loaded on the Chromium Controller (10× Genomics Inc.).

### Preparation of the 10× Genomics library

The cells were captured, and the genomics library was prepared using the Chromium Next GEM Single Cell 3′ GEM, Library & Gel Bead Kit v3.1, 16 rxns (#1000121; 10× Genomics) according to the manufacturer’s instructions. In brief, 5000 cells were targeted for capture per sample. After cDNA synthesis and amplification, the libraries were size-selected, pooled, and sequenced (2 × 150 bp paired-end sequencing) (Read1 [28 bp] + i7 index [8 bp] + i5 index [0 bp] + Read2 [91 bp] per lane with ~350 M paired reads per lane and 1 lane per sample) on the DNBSEQ-G400RS instrument (MGI). De-multiplexed raw sequencing reads were mapped to the mouse reference genome (mm10) using Cell Ranger software (v6.0.2) with the default parameters. The quality control metrics are listed in Supplementary Table 3. By using the R package Seurat (v.4d), gene expression values in individual data were log normalized by using the “NormalizeData” function and then integrated into a single dataset by using “IntegrateData”^58,59^. Genes detected in fewer than three cells were omitted from the analysis. To ensure the quality of the single-cell data and to remove the duplicates, we filtered the cells based on the number of detected features (nFeature_RNA) and mitochondrial content (percent.mt), based on the thresholds of nFeature_RNA (1000–4500) and percent.mt (<1%). Principal component analyses (PCAs) were performed on the detected genes for dimension reduction, and twenty PCAs were performed. We used the nearest-neighbor method to cluster the cells. Uniform Manifold Approximation and Projection (UMAP) was employed to identify the cell clusters^35,60^, with 0.4 resolution. Differential gene expression between clusters and samples (*Sox17-*cKO vs Controls) was determined by the Wilcoxon rank-sum test, by using “FindMarkers” or “FindAllMarkers” function. The dataset analyzed through the Seurat package was converted to the “Anndata” object, and then further analyses were performed using the Scanpy python module (v.1.9.1)^61^. We reran PCAs and the nearest-neighbor method and visualized the correlation of the cell clusters by using partition-based graph abstraction (PAGA)^62^ in the Scanpy module. The correlation heatmap was calculated using “scanpy.pl.correlation_matrix” function. The similarity between cell clusters was determined using Pearson’s correlation analysis, and attached hierarchical clustering was generated using ward linkage with 20 PCs. All scRNA-seq datasets have been deposited in the National Center for Biotechnology Information Gene Expression Omnibus (GSE 190043).

### Morphometric and statistical analyses

The geometry of the SV was assessed in terms of the valve leaflet angle. The angle between the cytoplasmic process of the Sertoli cells facing the RT lumen and the surface of the RT epithelial cells adjoining the Sertoli cells was taken as the valve leaflet angle and measured in H&E-stained sections (Fig. 1c). The number of Sertoli cells in the SV region was analyzed; SOX9 immunoreactive cells located within the seminiferous epithelia of the SV were counted (*i.e.,* 0–100 μm from the edge of the RT) (Figs. 1d, 7d). The edge of the RT was distinguished based on the morphology (RT epithelial cells are simple cuboidal cells, whereas Sertoli cells are tall columnar cells) or immunochemistry of RT markers, such as CDH1. Whole-mount immunohistochemistry was used to assess the germ cell distribution in the SV. The GFRα1^+^ A_undiff_ and SCP3^+^ spermatocytes located within the seminiferous epithelia of the SV (*i.e.,* 0–100 μm from the edge of the RT) were counted, as described previously^25^. Only the SV sectioned at the mid-sagittal plane was subjected to testicular section analysis. More than 50 cross-sectioned STs that appeared on the transverse testis section were analyzed per sample. The TUNEL^+^ apoptotic cells and SOX9^+^ Sertoli cells per cross-sectioned STs were counted^63^, and the tubule section area and height of the seminiferous epithelia were quantified using ImageJ software (v2.9.0; NIH). To quantify the SOX17^+^ and KI67^+^ RT epithelial cells, >500 RT epithelial cells were counted in each age group.

Quantitative data are presented as mean ± s.e.m. or as box-and-whisker plots, in which the median is shown as a line in the center of the box, and the whiskers show the range of sample distribution, excluding outliers. Data were analyzed using R (v.3.6.1; R Development Core Team) and Python (v.3.8.2) software. Two-tailed unpaired Student’s *t*-test was performed for single comparisons between two groups. For more than two groups, two-way repeated measures ANOVA with Bonferroni’s two-sided multiple comparisons was performed. Throughout this study, “*n*” refers to the number of animals. To ensure reproducibility, all the experiments were repeated three times (technical replicate) with similar results. All the experiments were performed with at least three distinct animals (biological replicate) with similar results. A *P*-value <0.05 was considered significant, and the levels of significance are represented as *P < 0.05, **P < 0.01.

### Diphtheria toxin-induced Sertoli cell ablation and Sertoli cell replacement

*AMH-Treck* mice^39^ was used to ablate the Sertoli cells throughout the testis. Since its diphtheria toxin receptor is fused with *eGFP*, endogenous Sertoli cells of *AMH-Treck* mice can be distinguished with GFP signals^39,40^. DT (Sigma-Aldrich) was reconstituted in DMSO and injected intraperitoneally into 3-week-old AMH-Treck mice (dose: 4 μg/kg), on 4 days prior to the Sertoli cell transplantation. Mouse testes were obtained from P7 *Sox17-*cKO mice or from their littermate control, and the cell suspension including immature Sertoli cells from the testes was prepared by two-step enzymatic digestion^25^. In brief, testes from 1-week-old (P7) *Sox17-*cKO and control mice were collected, rinsed extensively in HBSS, digested at 36 °C with a mixture of collagenase IV (1 mg/ml) and hydrogenase (1 mg/ml) for 15 min, and centrifuged at 300 × *g* for 1 min. The supernatant containing the interstitial cell population was discarded. The cells were digested in 0.25% Trypsin/EDTA for 15 min at 36 °C, and the reaction was inhibited with 10% FBS. DNase I was added at all digestion steps (1 mg/ml). The cells were washed in cold HBSS, filtered through a 40-μm cell strainer (Falcon), re-suspended in 10% FBS/Dulbecco’s Modified Eagle Medium with high glucose (Fujifilm) labeled with 0.4% trypan blue, and kept on ice until the transplantation. The resulting cell suspension at the concentration of 1.0 × 10^7^ cells/mL was then transplanted into the testes of DT-pretreated *AMH-Treck* recipients through the efferent ducts, under general anesthesia. In this study, the testes injected only with trypan blue solution was regarded as a sham control. All the testes were collected and subjected to analysis on day 45 after the transplantation. Most of the Sertoli cells of *AMH-Treck* mice were proven to be replaceable with donor-derived Sertoli cells in our previous study (the proportion of donor-derived Sertoli cells was shown to be 96.9 ± 0.5% at 45 days post-transplantation)^25^. GFP signals of the Sertoli cells of *AMH-Treck* mice were visualized by anti-GFP immunostaining due to the paraffin embedding process, which diminishes the endogenous GFP signals.

### Biotin tracer assay to monitor the integrity of the blood-testis barrier

EZ-Link™ Sulfo-NHS-LC-Biotin (Thermo Fisher) was diluted into PBS at the concentration of 10 mg/ml and injected into the testicular interstitium of 8-week-old *Sox17-*cKO mice and their littermate controls through the tunica albuginea (100μl per testis) by using a 31 G insulin syringe (Terumo Japan) under the general anesthesia^64^. The mice were euthanized 30 min after the injection, and their testes were snapped freezing, cryo-sectioned, and subjected to subsequent analysis. The distribution of the biotin tracer in the testis sections was visualized by using Alexa Fluor 488-streptavidin at a dilution of 1:250 for 30 min at room temperature^64^.

### Reporting summary

Further information on research design is available in the Nature Portfolio Reporting Summary linked to this article.

## Supplementary information

Supplementary Information

Description of Additional Supplementary Files

Supplementary Data 1

Supplementary Data 2

Supplementary Data 3

Supplementary Data 4

Reporting Summary

## Data Availability

All scRNA-seq datasets in this study have been deposited in the National Center for Biotechnology Information Gene Expression Omnibus (GSE190043). All data underlying the findings are fully available within the paper and its Supplementary data. Source data are provided with this paper.

## Source data

Source Data

## References

[1] de Rooij DG, Russell LD (2000). All you wanted to know about spermatogonia but were afraid to ask. J. Androl..

[2] Meng X (2000). Regulation of cell fate decision of undifferentiated spermatogonia by GDNF. Science.

[3] Takashima S (2015). Functional differences between GDNF-dependent and FGF2-dependent mouse spermatogonial stem cell self-renewal. Stem Cell Rep..

[4] Masaki K (2018). FGF2 has distinct molecular functions from GDNF in the mouse germline niche. Stem Cell Rep..

[5] Kitadate Y (2019). Competition for mitogens regulates spermatogenic stem cell homeostasis in an open niche. Cell Stem Cell.

[6] Ikami K (2015). Hierarchical differentiation competence in response to retinoic acid ensures stem cell maintenance during mouse spermatogenesis. Development.

[7] Nakamura Y (2021). Transient suppression of transplanted spermatogonial stem cell differentiation restores fertility in mice. Cell Stem Cell.

[8] Lord T, Oatley MJ, Oatley JM (2018). Testicular architecture is critical for mediation of retinoic acid responsiveness by undifferentiated spermatogonial subtypes in the mouse. Stem Cell Rep..

[9] Endo T, Freinkman E, de Rooij DG, Page DC (2017). Periodic production of retinoic acid by meiotic and somatic cells coordinates four transitions in mouse spermatogenesis. Proc. Natl Acad. Sci. USA..

[10] Griswold MD (2016). Spermatogenesis: the commitment to meiosis. Physiol. Rev..

[11] Bowles J (2006). Retinoid signaling determines germ cell fate in mice. Science.

[12] Anderson EL (2008). Stra8 and its inducer, retinoic acid, regulate meiotic initiation in both spermatogenesis and oogenesis in mice. Proc. Natl Acad. Sci. USA.

[13] O’Donnell L (2015). Mechanisms of spermiogenesis and spermiation and how they are disturbed. Spermatogenesis.

[14] Kulibin AY, Malolina EA (2021). The rete testis: development and role in testis function. Russ. J. Dev. Biol..

[15] Major AT, Estermann MA, Smith CA (2021). Anatomy, endocrine regulation, and embryonic development of the rete testis. Endocrinology.

[16] Kulibin AY, Malolina EA (2019). Formation of the rete testis during mouse embryonic development. Dev. Dyn..

[17] Omotehara T, Wu X, Kuramasu M, Itoh M (2020). Connection between seminiferous tubules and epididymal duct is originally induced before sex differentiation in a sex-independent manner. Dev. Dyn..

[18] Mayère C (2022). Origin, specification and differentiation of a rare supporting-like lineage in the developing mouse gonad. Sci. Adv..

[19] Garcia-Alenso L (2022). Single-cell roadmap of human gonadal development. Nature.

[20] Malolina EA, Kulibin AY (2019). The rete testis harbors Sertoli-like cells capable of expressing DMRT1. Reproduction.

[21] Geng X, Cha B, Mahamud MR, Srinivasan RS (2017). Intraluminal valves: development, function and disease. Dis. Model Mech..

[22] Bazigou E, Makinen T (2013). Flow control in our vessels: vascular valves make sure there is no way back. Cell Mol. Life Sci..

[23] Freck D (2021). ATP activation of peritubular cells drives testicular sperm transport. Elife.

[24] Aiyama Y (2015). A niche for GFRα1-positive spermatogonia in the terminal segments of the seminiferous tubules in hamster testes. Stem Cells.

[25] Imura-Kishi K (2021). Low retinoic acid levels mediate regionalization of the Sertoli valve in the terminal segment of mouse seminiferous tubules. Sci. Rep..

[26] Figueiredo AFA (2021). Insights into differentiation and function of the transition region between the seminiferous tubule and rete testis. Differentiation.

[27] Nagasawa K (2018). Regionally distinct patterns of STAT3 phosphorylation in the seminiferous epithelia of mouse testes. Mol. Reprod. Dev..

[28] Roosen-Runge EC (1961). The rete testis in the albino rat: its structure, development and morphological significance. Acta Anatomica.

[29] Tainosho S (2011). Multilayered structure of the basal lamina of the tubuli recti in normal mice. Med Mol. Morphol..

[30] Corada M (2013). Sox17 is indispensable for acquisition and maintenance of arterial identity. Nat. Commun..

[31] Liu M (2019). Sox17 is required for endothelial regeneration following inflammation-induced vascular injury. Nat. Commun..

[32] Nykänen M, Kormuno M (1978). Early effects of efferent duct ligation on the rat rete testis. Int. J. Androl..

[33] Yan HH, Mruk DD, Lee WM, Cheng CY (2007). Ectoplasmic specialization: a friend or a foe of spermatogenesis?. Bioessays.

[34] Osman DI (1978). On the ultrastructure of modified Sertoli cells in the terminal segment of seminiferous tubules in the boar. J. Anat..

[35] Osman DI, Ploen L (1978). The mammalian tubuli recti: ultrastructural study. Anat. Rec..

[36] Becht E (2018). Dimensionality reduction for visualizing single-cell data using UMAP. Nat. Biotechnol..

[37] Bellvé AR (1977). Spermatogenic cells of the prepuberal mouse. Isolation and morphological characterization. J. Cell Biol..

[38] McGregor GR (1990). Symplastic spermatids (sys): a recessive insertional mutation in mice causing a defect in spermatogenesis. Proc. Natl Acad. Sci. U. S. A..

[39] Shinomura M (2014). A novel Amh-Treck transgenic mouse line allows toxin-dependent loss of supporting cells in gonads. Reproduction.

[40] Higuchi K (2021). Sertoli cell replacement in explanted mouse testis tissue supporting host spermatogenesis. Biol. Reprod..

[41] Kanazawa Y, Omotehara T, Nakata H, Hirashima T, Itoh M (2022). Three-dimensional analysis and in vivo imaging for sperm release and transport in the murine seminiferous tubule. Reproduction.

[42] Fisher, New light shed on fluid formation in the seminiferous tubules of the rat. *J. Physiol.* **542**, 445–452 (2002). 10.1113/jphysiol.2002.018648 PMC2290430 12122144

[43] Tuck RR, Setchell BP, Waites GMH, Young JA (1970). The composition of fluid collected by micropuncture and catheterization from the seminiferous tubules and rete testis of rats. Pflügers Arch..

[44] Yan (1999). Stage-specific regulation of stem cell factor gene expression in the rat seminiferous epithelium. Endocrinology.

[45] Azhar M (2012). Transforming growth factor Beta2 is required for valve remodeling during heart development. Dev. Dyn..

[46] Lu P (2022). A SOX17-PDGFB signaling axis regulates aortic root development. Nat. Commun..

[47] Zhang Y (2009). Non-Smad pathways in TGF-beta signaling. Cell Res..

[48] Hamidi A (2017). TGF-β promotes PI3K-AKT signaling and prostate cancer cell migration through the TRAF6-mediated ubiquitylation of p85α. Sci. Signal.

[49] Binnerts ME (2007). R-Spondin1 regulates Wnt signaling by inhibiting internalization of LRP6. Proc. Natl Acad. Sci. U. S. A..

[50] Dubey R (2020). R-spondins engage heparan sulfate proteoglycans to potentiate WNT signaling. Elife.

[51] Tanwar PS (2010). Constitutive WNT/beta-catenin signaling in murine Sertoli cells disrupts their differentiation and ability to support spermatogenesis. Biol. Reprod..

[52] Barrionuevo F, Scherer G (2010). SOX E genes: SOX9 and SOX8 in mammalian testis development. Int J. Biochem Cell Biol..

[53] Hirate Y (2016). Mouse Sox17 haploinsufficiency leads to female subfertility due to impaired implantation. Sci. Rep..

[54] Dhillon H (2006). Leptin directly activates SF1 neurons in the VMH, and this action by leptin is required for normal body-weight homeostasis. Neuron.

[55] Spence JR (2009). Sox17 regulates organ lineage segregation of ventral foregut progenitor cells. Dev. Cell.

[56] Kim I, Saunders TL, Morrison SJ (2007). Sox17 dependence distinguishes the transcriptional regulation of fetal from adult hematopoietic stem cells. Cell.

[57] Takase Y, Tadokoro R, Takahashi Y (2013). Low-cost labeling with highlighter ink efficiently visualizes developing blood vessels in avian and mouse embryos. Dev. Growth Differ..

[58] Stuart T (2019). Comprehensive integration of single-cell data. Cell.

[59] Hao Y (2021). Integrated analysis of multimodal single-cell data. Cell.

[60] Yamada Y (2021). Single-cell transcriptional analysis reveals developmental stage-dependent changes in retinal progenitors in the murine early optic vesicle. Biochem. Biophys. Res. Commun..

[61] Wolf F, Angerer P, Theis F (2018). SCANPY: large-scale single-cell gene expression data analysis. Genome Biol..

[62] Wolf FA (2019). PAGA: graph abstraction reconciles clustering with trajectory inference through a topology preserving map of single cells. Genome Biol..

[63] Uchida A (2020). Development and function of smooth muscle cells is modulated by Hic1 in mouse testis. Development.

[64] Chen H (2018). Monitoring the integrity of the blood-testis barrier (BTB): an in vivo assay. Mehods Mol. Biol..

