# Supplementary material for: SOX17-positive rete testis epithelium is required for Sertoli valve formation and normal spermiogenesis in the male mouse
Source: Nat Commun. 2022 Dec 21;13:7860. doi: 10.1038/s41467-022-35465-1 (PMC9772346; doi:10.1038/s41467-022-35465-1)
Supplement: Supplementary file 1 — Supplementary Information [file 41467_2022_35465_MOESM1_ESM.pdf]

Supplementary figures and figure legends

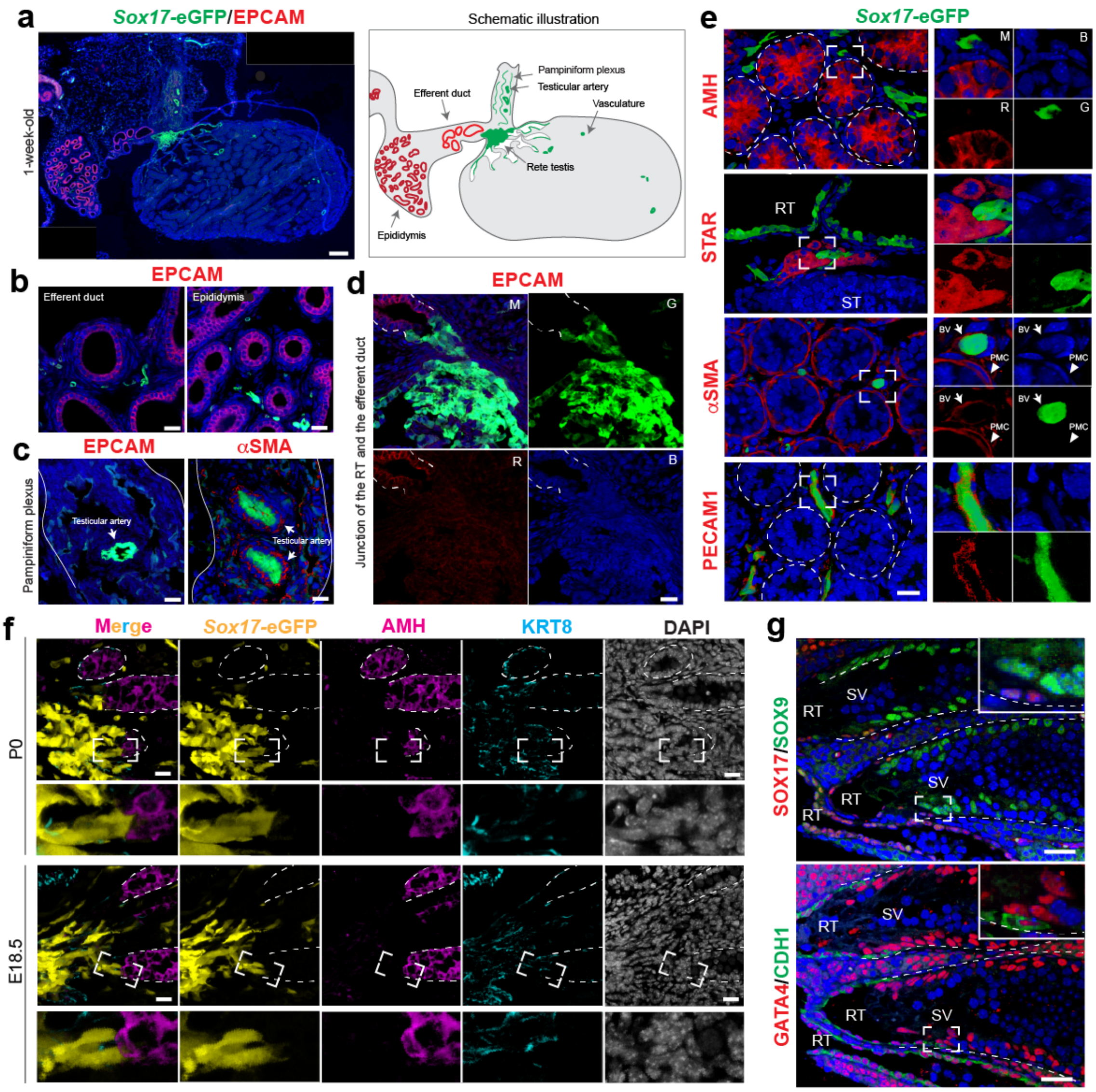

Supplementary Fig. 1 | SOX17 expression in the RT epithelial cells. a–e, *Sox17*-eGFP (green) signals in the testis of a 1-week-old *Sox17*$^{+/eGFP}$ mouse, showing the expression of *Sox17*-eGFP in the RT. **a,** Low magnification of the longitudinal testicular section. The right panel shows a schematic illustration of the image shown in the left panel, illustrating the anatomical structure around the cranial pole of the testis. *Sox17*-eGFP (green) signals were observed in the RT and the intra-/extra-testicular vasculature, but not in the efferent duct and epididymis of which epithelium are marked by EPCAM immunoreactivity (red). **b,** Epithelial cells of the efferent duct and epididymis marked by the EPCAM do not express *Sox17*-eGFP. **c,** The testicular artery and the surrounding pampiniform plexus (αSMA^{+}/EPCAM^{-}) express *Sox17*-eGFP. **d,** Junctional region of the RT (EPCAM^{low}) and the efferent duct (EPCAM^{high}). *Sox17*-eGFP expresses distinctly in the RT epithelium. **e,** Sertoli cells (AMH^{+}), Leydig cells (STAR^{+}), and peritubular myoid cells (αSMA^{+}; arrows) do not express *Sox17*-eGFP. On the other hand, *Sox17*-eGFP signals were observed in vascular endothelial cells residing in the testicular interstitium, which are distinguished by PECAM-1 and αSMA signals (arrowheads). **f,** The SV region of the postnatal (upper panels, p0) and prenatal testis (lower panels, E18.5) of *Sox17*$^{+/eGFP}$ mouse. Sertoli cells at the SV region marked by AMH (magenta) are negative for *Sox17*-eGFP (yellow) and KRT8 (cyan). **g,** The SV region of 4-week-old wild-type mouse testis, immuno-stained by SOX9, SOX17, GATA4, and CDH1. The sections shown in the top and the bottom panel are serial to each other. SOX17-positive RT epithelial cells are also positive for CDH1 and GATA4. Right panels in e show magnification of the region surrounded by a broken square. Lower panels in f show magnification of the region surrounded by a broken rectangle in the upper panel. Inset in g shows the magnification of the region surrounded by a broken rectangle in each panel. **d** and the right panels in **e** show merged picture (“M”), together with isolated channels of blue (“B”), Red (“R”), and green (“G”) color. Figures in f show a merged channel (left-most panel), with yellow, magenta, cyan, and white channel on the right. Arrows, SOX17 immunoreactivity in vascular endothelial cells; Arrowheads, peritubular myoid cells; White sold lines, outlines of the pampiniform plexus. Broken line, outlines of the seminiferous tubules; BV, blood vessel; RT, rete testis; PMC, peritubular myoid cell; ST, Seminiferous tubule; SV, Sertoli valve. The experiments were performed with at least three distinct animals with similar results. Scale bars, 200 μm (**a**), 20 μm (**b–g**).

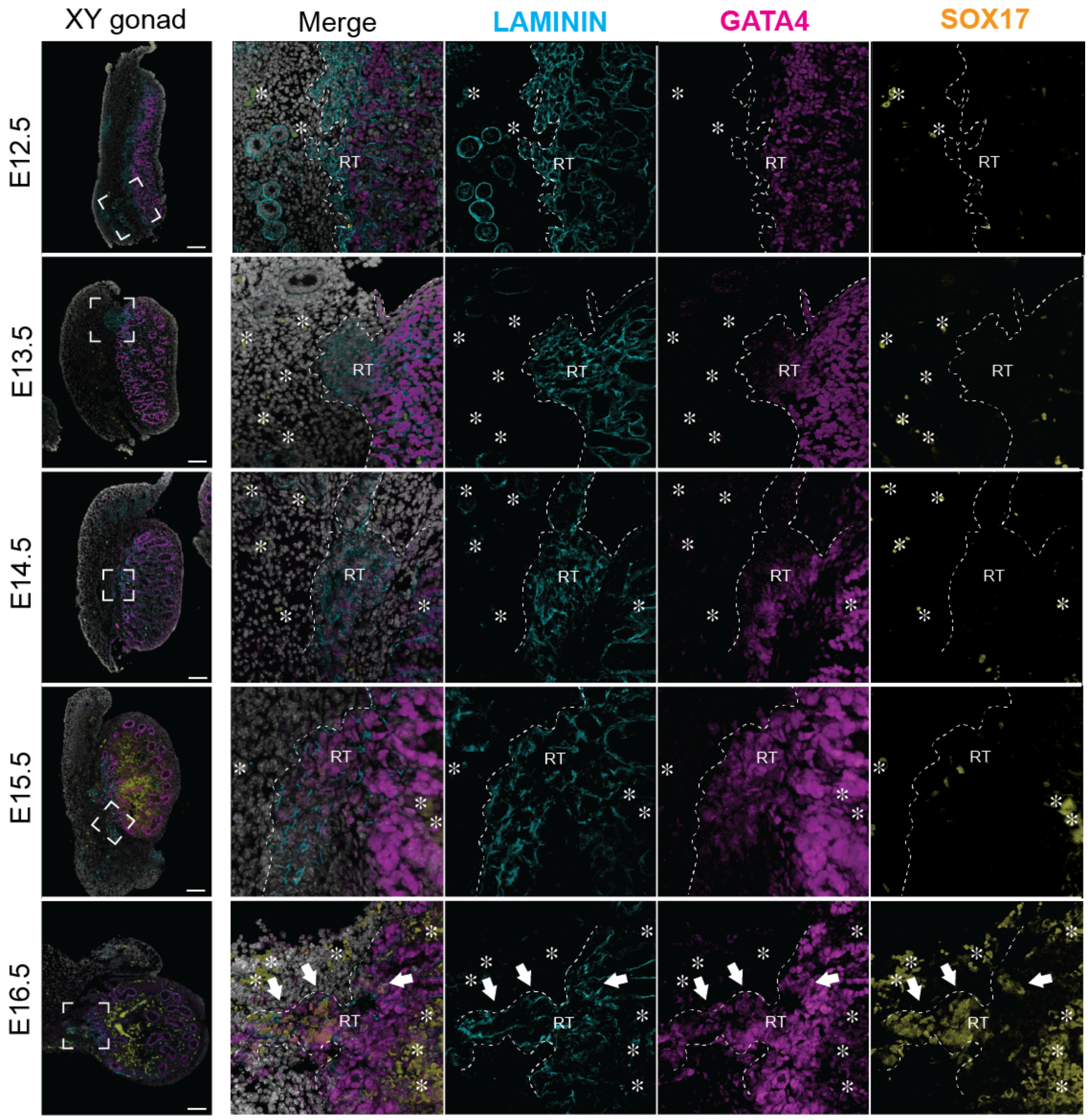

Supplementary Fig. 2 | SOX17 expression in the RT of embryonic XY gonads. Sections of wild-type XY embryonic gonads at E12.5, E13.5, E14.5, E15.5, and E16.5, immuno-stained with LAMININ (cyan, basement membrane), GATA4 (magenta, testicular somatic cells), and SOX17 (yellow). A broken square in the leftmost panel indicates regions shown as merged and isolated channels on the right panels. SOX17 expression in GATA4$^+$ somatic cells located in the RT region becomes evident from E16.5 onwards. Asterisks, autofluorescence in erythrocytes, and non-specific signals in the interstitium; Arrows; The RT with GATA4$^+$ somatic cells that express SOX17. Broken line, border of the RT region; RT; rete testis. The experiments were performed with at least three distinct animals with similar results. Scale bars, 100 μm.

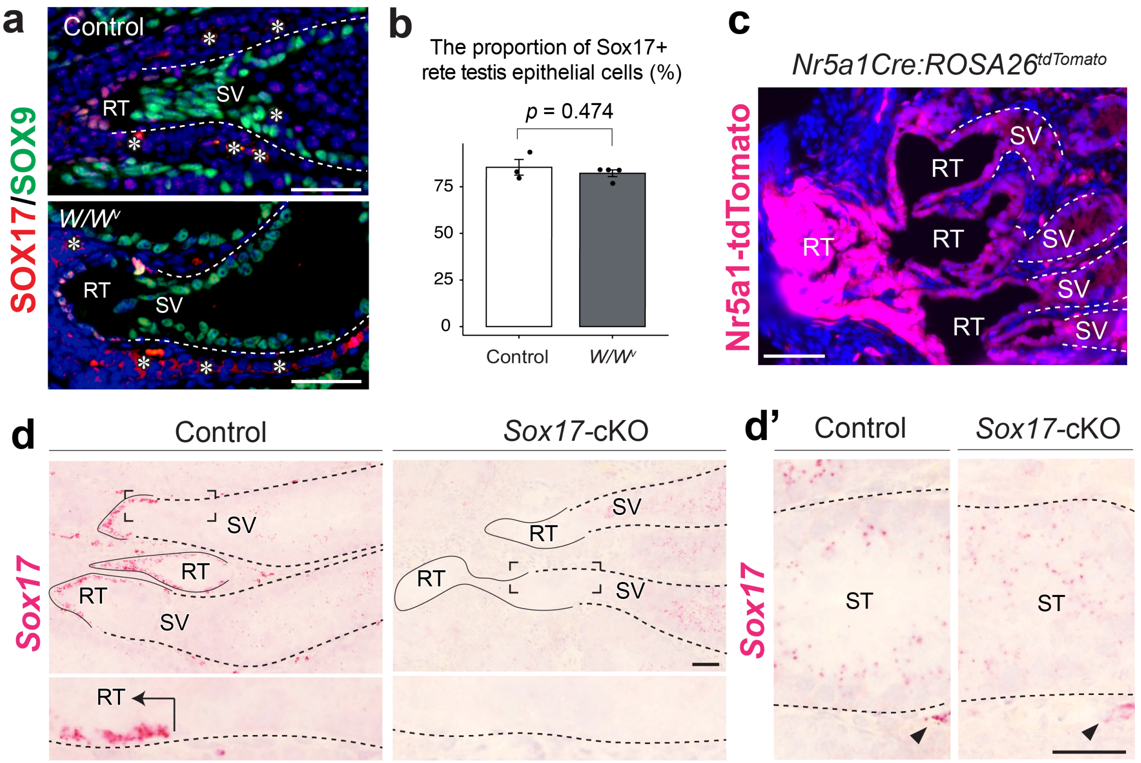

Supplementary Fig. 3 | The expression of Sox17 in the RT and its ablation in *Sox17*-cKO mice. a, Anti-SOX17 and SOX9 immunostaining of the proximal part of the testis from germ cell-deficient W/W^{v} mutant mice and wild-type mice at 4 weeks of age. The expression of SOX17 in the RT was conserved in W/W^{v} mutant mice. **b,** the proportion of $SOX17^{+}$ RT epithelial cells at 4 weeks of age did not significantly differ depending on the presence of germ cells. Control n = 3, W/W^{v} n = 4. **c,** Expression of *tdTomato* in the RT epithelia from 1-week-old *Nr5a1-Cre:* $ROSA26^{tdTomato}$ mice. **d,** RNA *in situ* hybridization of the control and *Nr5a1Cre:* $Sox17^{flox/flox}$ (*Sox17*-cKO) mouse testes at 4 weeks of age showing reduced *Sox17* signals in the RT epithelia of *Sox17*-cKO testes. Meanwhile, its expression in germ cells in the ST and vasculature in the interstitium (arrows) were conserved in *Sox17*-cKO mice (**d’**). The lower panels in d show magnified images of the region indicated by the broken rectangles in the upper panels. Data are mean ± s.e.m. Comparisons were made using a two-tailed unpaired Student’s *t*-test. Asterisk, autofluorescence in erythrocytes; Arrow, a border of the RT; Arrowheads, Sox17 signals in vascular endothelial cells; Broken lines, tubular wall; RT, rete testis; ST, Seminiferous tubule; SV, Sertoli valve. “n” represents the number of biological replicates,

and the experiments were performed with at least three distinct animals with similar results. Scale bars, 50μm.

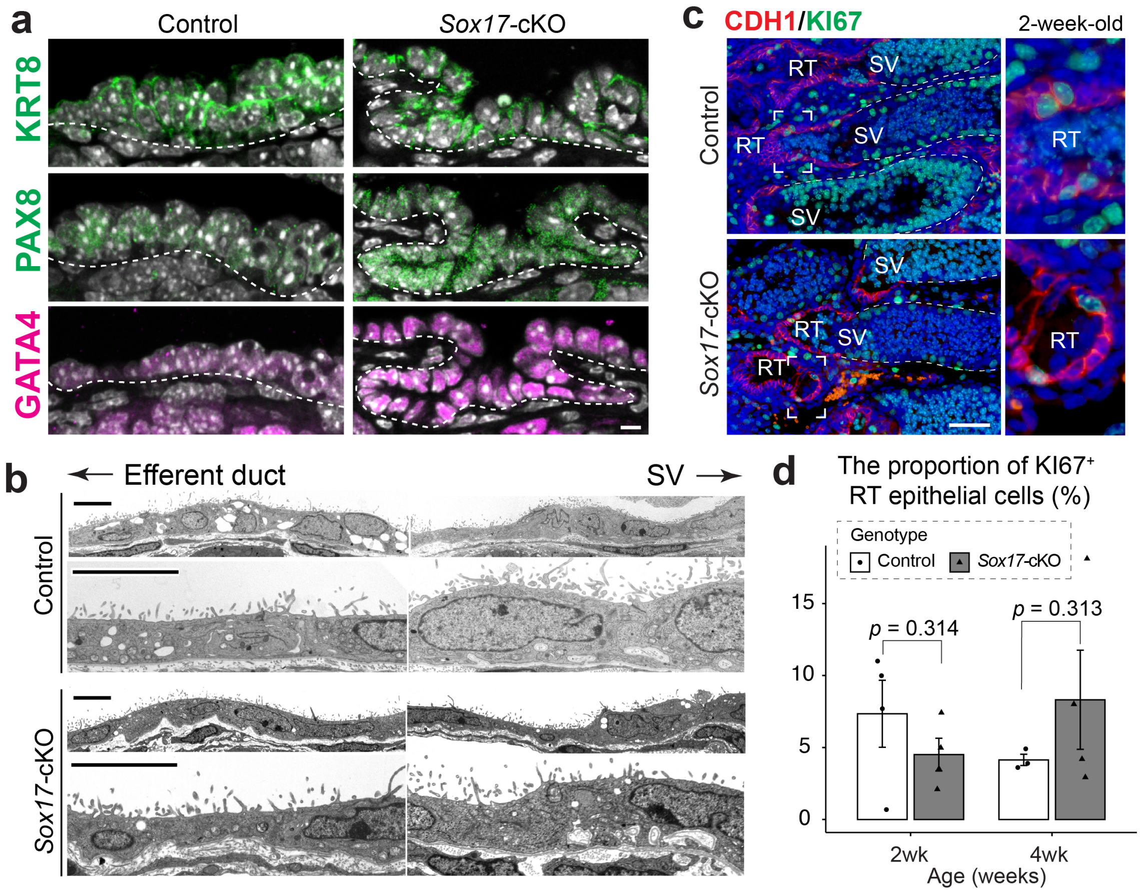

Supplementary Fig. 4 | Little morphological defects were observed in *Sox17*-depleted RT epithelia. a, KRT8, PAX8, and GATA4 immunostaining in serial sections of the RT, showing no apparent changes in marker expression in *Sox17*-depleted RT epithelia. **b,** Transmission electron microscopes (TEM) of the RT epithelia from 4-week-old *Sox17*-cKO mice showing no apparent morphological abnormalities in the ultrastructure. The left panels represent the proximal RT region close to the efferent duct, while the right panels show the distal RT region near the SV. **c,** KI67 and CDH1 immunostaining of the RT epithelia from 2-week-old *Sox17*-cKO mice and their littermate controls. **d,** the proportion of $Ki67^{+}$ proliferative RT epithelial cells remained similar between *Sox17*-cKO and the controls at 2 and 4 weeks of age. 2wk: Control n = 4, *Sox17*-cKO n=4; 4wk: Control n = 3, *Sox17*-cKO n=4. The right panels in **c** represent magnified images of the region surrounded by broken rectangles on the left. Data are mean ± s.e.m. Comparisons were made using a two-tailed unpaired Student's *t*-test (**d**). Broken line, tubular wall; RT, rete testis, SV, Sertoli valve. "n" represents the number of

biological replicates, and the experiments were performed with at least three distinct animals with similar results. Scale bars, 5 μm (**a, b**), 50 μm (**c, e**).

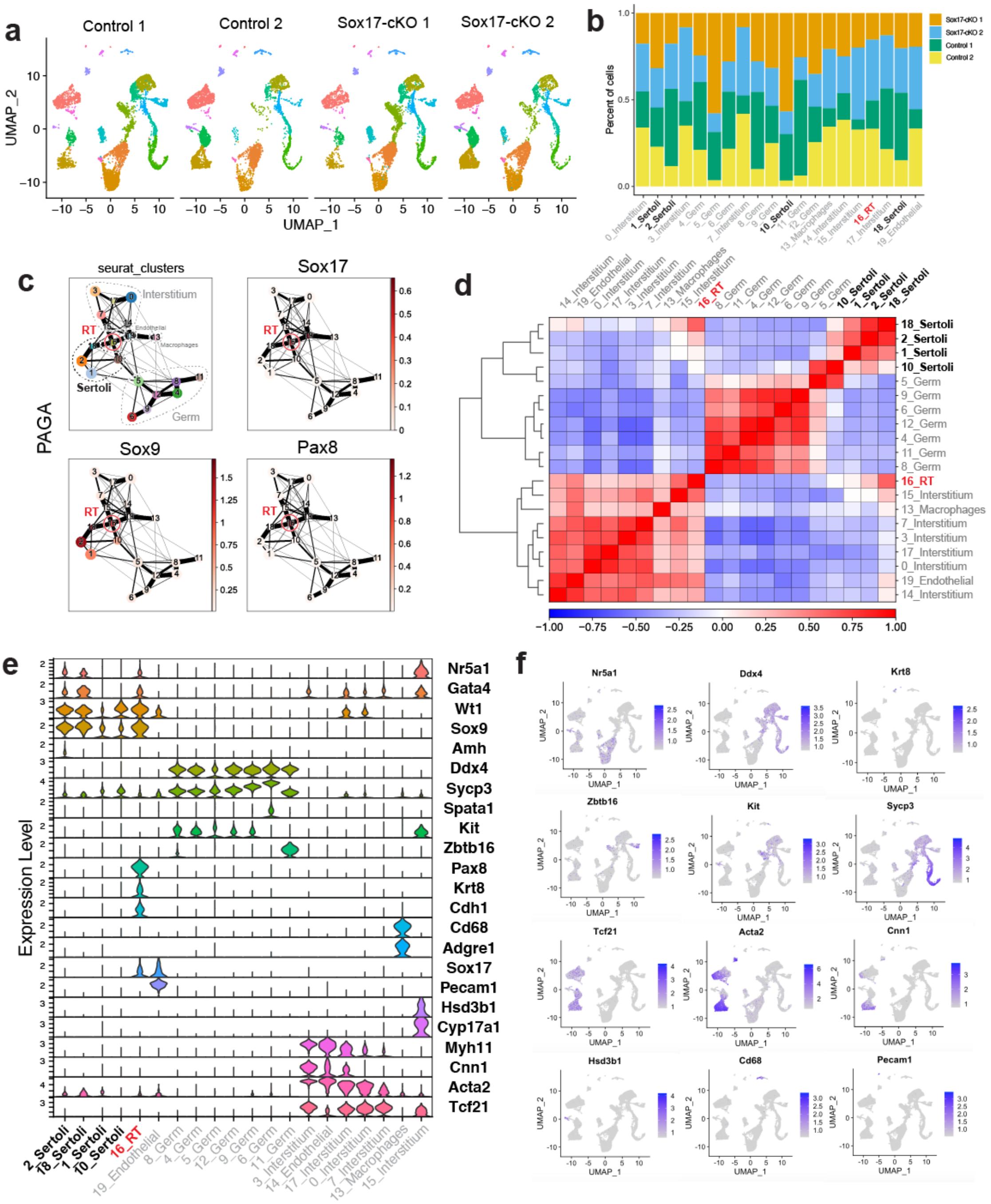
a
Control 1
Control 2
Sox17-cKO 1
Sox17-cKO 2
UMAP_2
UMAP_1
b
Percent of cells
Sox17-cKO 1
Sox17-cKO 2
Control 1
Control 2
0_Interstitium
1_Sertoli
2_Sertoli
3_Interstitium
4_Germ
5_Germ
6_Germ
7_Interstitium
8_Germ
9_Germ
10_Sertoli
11_Germ
12_Germ
13_Macrophages
14_Interstitium
15_Interstitium
16_RT
17_Interstitium
18_Sertoli
19_Endothelial
c
PAGA
seurat_clusters
Interstitium
RT
Endothelial
Macrophages
Sertoli
Germ
Sox17
Sox9
Pax8
d
18_Sertoli
2_Sertoli
1_Sertoli
10_Sertoli
5_Germ
9_Germ
6_Germ
12_Germ
4_Germ
11_Germ
8_Germ
16_RT
15_Interstitium
13_Macrophages
7_Interstitium
3_Interstitium
17_Interstitium
0_Interstitium
19_Endothelial
14_Interstitium
−1.00
−0.75
−0.50
−0.25
0.00
0.25
0.50
0.75
1.00
e
Expression Level
Nr5a1
Gata4
Wt1
Sox9
Amh
Ddx4
Sycp3
Spata1
Kit
Zbtb16
Pax8
Krt8
Cdh1
Cd68
Adgre1
Sox17
Pecam1
Hsd3b1
Cyp17a1
Myh11
Cnn1
Acta2
Tcf21
f
Nr5a1
Ddx4
Krt8
Zbtb16
Kit
Sycp3
Tcf21
Acta2
Cnn1
Hsd3b1
Cd68
Pecam1
UMAP_1
UMAP_2

Supplementary Fig. 5 | Cluster identification in the scRNA-seq data. a, UMAP plots of an individual sample, represented separately. Two independent replicates per genotype were analyzed in the scRNA-seq analysis in this study. **b,** Proportion of cells that contributed to each cluster by genotype. **c,** PAGA representation of the clusters, illustrating the expression of *Sox17*, *Sox9*, and *Pax8*. Each node represents a cluster, and the lines connecting two clusters represent the confidence of the relation between the clusters. **d,** Pearson correlation map of the identified clusters, showing the distinct transcriptome profiles of the RT and Sertoli cells. **e,** Violin plots representing the expression levels of representative marker genes for each cell type. **f,** UMAP plots showing the distribution of representative marker genes for each cell type**.** The identity of clusters is as follows. The rete testis epithelial cells: cluster 16. Sertoli cells: clusters 1, 2, 10, 18. Germ cells: clusters 4–6, 8–9, 11–12. Interstitium: clusters 0, 3, 7, 15, 17. Macrophages: cluster 13. Vascular endothelial cells: cluster 19.

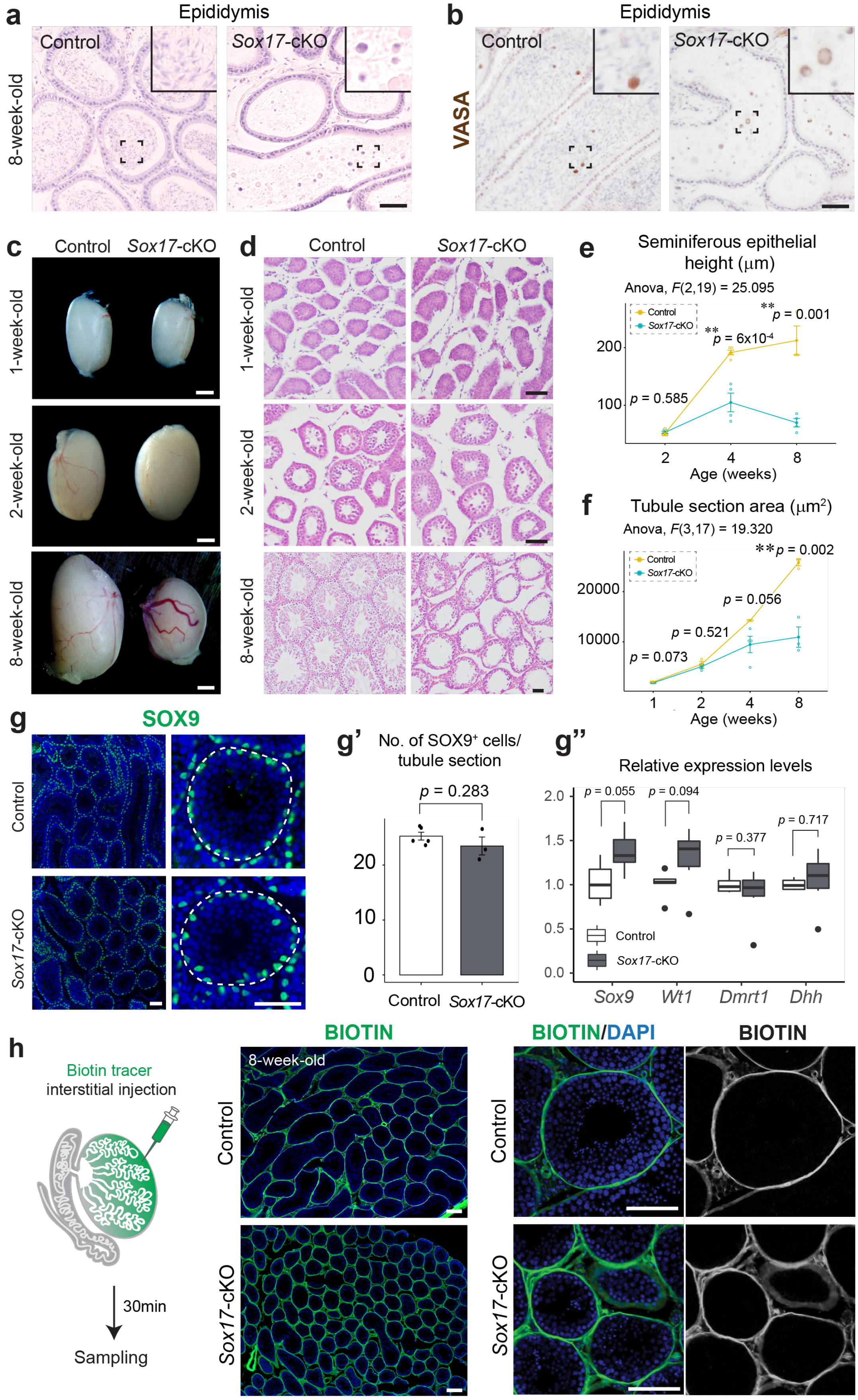

a
Epididymis
8-week-old
Control
Sox17-cKO
b
Epididymis
VASA
Control
Sox17-cKO
c
Control Sox17-cKO
1-week-old
2-week-old
8-week-old
d
Control Sox17-cKO
1-week-old
2-week-old
8-week-old
e
Seminiferous epithelial height (μm)
Anova, F(2,19) = 25.095
Control
Sox17-cKO
** p = 0.001
** p = 6x10-4
p = 0.585
200
100
2 4 8
Age (weeks)
f
Tubule section area (μm2)
Anova, F(3,17) = 19.320
Control
Sox17-cKO
** p = 0.002
p = 0.056
p = 0.521
p = 0.073
20000
10000
1 2 4 8
Age (weeks)
g
SOX9
Control
Sox17-cKO
g'
No. of SOX9+ cells/ tubule section
p = 0.283
20
10
0
Control Sox17-cKO
g''
Relative expression levels
p = 0.055
p = 0.094
p = 0.377
p = 0.717
2.0
1.5
1.0
0.5
0.0
Control
Sox17-cKO
Sox9 Wt1 Dmrt1 Dhh
h
Biotin tracer interstitial injection
30min
Sampling
BIOTIN
8-week-old
Control
Sox17-cKO
BIOTIN/DAPI
BIOTIN
Control
Sox17-cKO

Supplementary Fig. 6 | Progressively severe spermatogenic defects in post-pubertal *Sox17*-cKO mice. a–b, H&E staining (a) and anti-VASA immunostaining (b) of the epididymis section from control and *Sox17*-cKO littermates at 8 weeks of age. The epididymis of *Sox17*-cKO mice contained hardly any spermatozoa, while some immature germ cells marked by VASA were observed in the epididymal duct. **c–f.** Phenotypic characterization of the ST in *Sox17*-cKO mice and their littermate controls at 1, 2, and 8 weeks old. Gross morphology of the testis (**b**) and H&E staining of the transverse testis section (**c**) showing no appreciable spermatogenic defects in the ST by 2 weeks of age in *Sox17*-cKO mice. Post-pubertal spermiogenic defects in *Sox17*-cKO mice lead to progressive degeneration of the seminiferous epithelia in STs by 8 weeks of age (**e, f**). e: Control n=5, *Sox17*-cKO n=4, 4wk: Control n=4, *Sox17*-cKO n=5, 8wk: Control n=4, *Sox17*-cKO n=3. f: 1wk: Control n=3, *Sox17*-cKO n=3, 2wk: Control n=3, *Sox17*-cKO n=3, 4wk: Control n=4, *Sox17*-cKO n=3, 8wk: Control n=3, *Sox17*-cKO n=3. **f,** Control n=5, *Sox17*-cKO n=3. **g.** Distribution of Sertoli cells in the ST of 4-week-old *Sox17*-cKO mice and controls. SOX9 immunostaining shows that the distribution of Sertoli cells marked by SOX9 was not disrupted in *Sox17*-cKO mice. The number of Sertoli cells in each cross-sectioned ST was consistent between *Sox17*-cKO and control animals (**g'**). Control n=6, *Sox17*-cKO n=4. g'' RT-qPCR analysis of the whole testis from 4-week-old *Sox17*-cKO and control group animals show that the expression levels of Sertoli cell-specific marker genes were not significantly altered in *Sox17*-cKO mice. Control n=4, *Sox17*-cKO n=6 for *Sox9* and Control n=6, *Sox17*-cKO n=6 for others. **h,** Immunohistochemistry of streptavidin AlexaFluor488 conjugate on the testis of 8-week-old control and *Sox17*-cKO mice injected with Biotin tracer into the interstitium, together with a schematic of the experiment on the left. The left panels show low images of the testis at low magnification, while the right panels show the high magnification image with a single-colored channel of the biotin tracer. No biotin tracer was observed in the adluminal compartment of the seminiferous tubules both in the control and *Sox17*-cKO mice. The insets show magnified views of the region surrounded by broken rectangles in each panel (**a, b**). Broken line, tubular wall. Data are mean ± s.e.m (**e, f, g'**) or as box-and-whisker plots displaying median, interquartile range (boxes), and minima and maxima (whiskers) (**g''**). Comparisons were made using a two-tailed unpaired Student's *t*-test (**f'**) or two-way repeated-measures ANOVA with Bonferroni's two-sided multiple comparisons (**d, e**). "n" represents the number of biological replicates, and the

experiments were performed with at least three distinct animals with similar results. *$P<0.05$, **$P<0.01$. Scale bars, 50 μm (**a**–**b, d**–**h**), 1 mm (**c**).

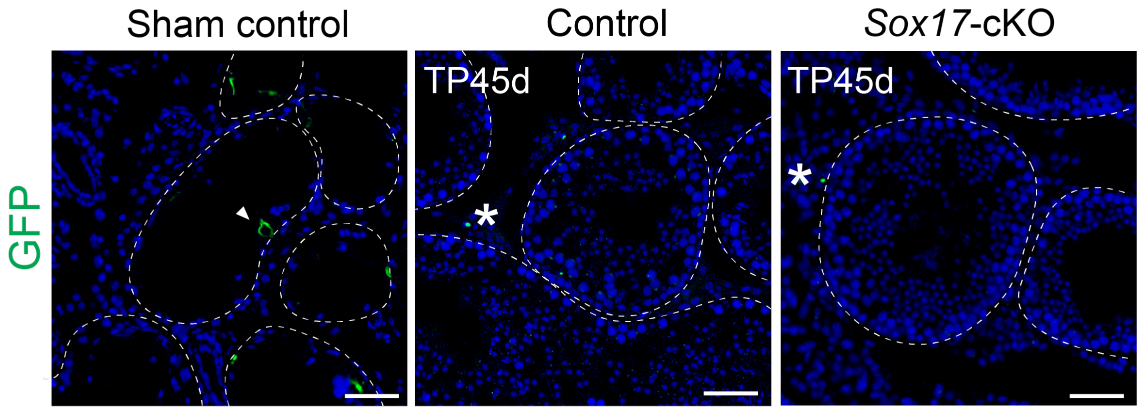

Supplementary Fig. 7 | GFP-negative donor-derived cells support spermatogenesis in the STs of *AMH-Treck* mouse testes at 45 days post-transplantation. Immunohistochemistry of GFP in paraffin-embedded testicular sections of diphtheria toxin-pretreated *AMH-Treck* mouse at 45 days after the transplantation. Endogenous Sertoli cells of *AMH-Treck* mice that remained in the ST of sham control testis were distinguished by GFP signals. Spermatogenesis recovered in the *AMH-Treck* testes transplanted with Sertoli and germ cells collected either from *Sox17*-cKO mice (*Sox17*-cKO) or their littermate wild-type control (Control) were supported by GFP^- donor-derived cells. In this analysis, GFP signals were visualized with GFP immunoreactivity owing to the paraffin embedding process, which diminishes the endogenous EGFP signals. Arrowheads, remaining endogenous Sertoli cells of *AMH-Treck* mice (GFP^+). Asterisk, autofluorescence in erythrocytes. Broken line, tubular wall. ST, convoluted seminiferous tubule. TP, post-transplantation. The experiments were performed with at least three distinct animals with similar results. Scale bars, 50 μm.

Supplementary Tables

Supplementary Table 1 | List of probes used for *in situ* hybridization

Gene symbol	CAT#	Accession No.
Fgf9	499811	NM_013518.4
Rspo1	401991	NM_138683.2
Tgfb2	406181	NM_009367.3
Wnt4	401109	NM_009523.2
Sox17	493151	NM_011441.5
PpiB (positive control)	313911	NM_011149.2
DapB (Negative control)	310043	EF191515

Supplementary Table 2 | List of primers used for RT-qPCR

Target gene	Direction	Sequence (5' → 3')
Actb	F	CCTTCTTGGGTATGGAATCCTGT
	R	CACTGTGTTGGCATAGAGGTCTTTAC
Kit	F	AACAACAAAGAGCAAATCCAGG
	R	GGAAGTTGCGTCGGGTCTAT
Dhh	F	CGGGACCTCGTACCCAACTA
	R	TTGCAACGCTCTGTCATCAG
Dmrt1	F	AGAAGCCAAAGCCAGTGTGTTC
	R	ATTCCAGAACTCCCTTCGAGC
GFRα1	F	ACTGGAGCATGTACCAGAGC
	R	CTTTGGAAATGTGTTCCACTGA
Odf1	F	CCGCAGTTTAGAGAGACTCAG
	R	GATCAGGTTCAAAGCCGCAC
Pax7	F	GGCACAGAGGACCAAGCTC
	R	GCACGCCGGTTACTGAAC
Plzf	F	CGTTGGGGGTCAGCTAGAAAG
	R	CACCATGATGACCACATCGC
Prm1	F	ACAGCCCACAAAATTCCACC
	R	CTTATGGTGTATGAGCGGCG
Sall4	F	CGTTGACACCGAAGGTCGTA
	R	ATGCTAGCAAAGGCGGCATA
Scp3	F	ACACGAGCAGTTCATAAAGAG
	R	TGCCATCTCTTGCTGCTGAG
Sox9	F	AGGAAGTCGGTGAAGAACGG

	R	GGACCCTGAGATTGCCCAGA
Stra8	F	GGAGAAAAAGGCCAGACTCC
	R	CCACGTCAAAAGCATCTTCA
Wt1	F	CCAGTGTAAAACTTGTCAGCGAAA
	R	ATGAGTCCTGGTGTGGGTCTTC

Supplementary Table 3. Mapping results of samples analyzed by scRNA-seq

Samples	Total Reads	Mean reads/cell	Median genes/cell	No. of genes detected	No. of cells captured
Control 1	352267824	26359	1640	23779	13364
Control 2	376410173	40095	2804	23517	9388
Sox17-cKO *1*	355820148	29018	1368	23877	12262
Sox17-cKO *2*	355464304	32353	2153	23187	10987

Supplementary Data 1. Marker genes of clusters 0–20 in scRNA-seq analysis.

Supplementary Data 2. Differentially expressed genes in the *Sox17*-cKO RT compared to the control RT

Supplementary Data 3. Differentially expressed genes in Sertoli cell cluster 18 compared to Sertoli cell clusters 1, 2, and 10

Supplementary Data 4. Differentially expressed genes in Sertoli cell clusters (1, 2, 10, 18) between the *Sox17*-cKO and the controls.
