# Supplementary material for: SOX17-positive rete testis epithelium is required for Sertoli valve formation and normal spermiogenesis in the male mouse
Source: Nat Commun. 2022 Dec 21;13:7860. doi: 10.1038/s41467-022-35465-1 (PMC9772346; doi:10.1038/s41467-022-35465-1)
Supplement: Supplementary file 2 — Description of Additional Supplementary Files [file 41467_2022_35465_MOESM2_ESM.pdf]

Description of Additional Supplementary Files

Supplementary Data 1. Marker genes of clusters 0–20 in scRNA-seq analysis

Supplementary Data 2. Differentially expressed genes in the Sox17-cKO RT compared to the control RT

Supplementary Data 3. Differentially expressed genes in Sertoli cell cluster 18 compared to Sertoli cell clusters 1, 2, and 10

Supplementary Data 4. Differentially expressed genes in Sertoli cell clusters (1, 2, 10, 18) between the Sox17-cKO and the controls
