# Supplementary material for: SOX17-positive rete testis epithelium is required for Sertoli valve formation and normal spermiogenesis in the male mouse
Source: Nat Commun. 2022 Dec 21;13:7860. doi: 10.1038/s41467-022-35465-1 (PMC9772346; doi:10.1038/s41467-022-35465-1)
Supplement: Supplementary file 7 — Reporting Summary [file 41467_2022_35465_MOESM7_ESM.pdf]

nature portfolio

Corresponding author(s): Yoshiakira Kanai

Last updated by author(s): Nov 27, 2022

Reporting Summary

Nature Portfolio wishes to improve the reproducibility of the work that we publish. This form provides structure for consistency and transparency in reporting. For further information on Nature Portfolio policies, see our Editorial Policies and the Editorial Policy Checklist.

Statistics

For all statistical analyses, confirm that the following items are present in the figure legend, table legend, main text, or Methods section.

n/a	Confirmed	
☐	☒	The exact sample size (*n*) for each experimental group/condition, given as a discrete number and unit of measurement
☐	☒	A statement on whether measurements were taken from distinct samples or whether the same sample was measured repeatedly
☐	☒	The statistical test(s) used AND whether they are one- or two-sided<br>*Only common tests should be described solely by name; describe more complex techniques in the Methods section.*
☐	☒	A description of all covariates tested
☐	☒	A description of any assumptions or corrections, such as tests of normality and adjustment for multiple comparisons
☐	☒	A full description of the statistical parameters including central tendency (e.g. means) or other basic estimates (e.g. regression coefficient) AND variation (e.g. standard deviation) or associated estimates of uncertainty (e.g. confidence intervals)
☐	☒	For null hypothesis testing, the test statistic (e.g. *F*, *t*, *r*) with confidence intervals, effect sizes, degrees of freedom and *P* value noted<br>*Give P values as exact values whenever suitable.*
☒	☐	For Bayesian analysis, information on the choice of priors and Markov chain Monte Carlo settings
☒	☐	For hierarchical and complex designs, identification of the appropriate level for tests and full reporting of outcomes
☐	☒	Estimates of effect sizes (e.g. Cohen's *d*, Pearson's *r*), indicating how they were calculated

Our web collection on statistics for biologists contains articles on many of the points above.

Software and code

Policy information about availability of computer code

Data collection	Image data were obtained by using by fluorescence microscopy (BX51N-34-FL-2) or Leica TCS SP8 confocal laser microscopy.The the tubule section area and height of the seminiferous epithelia were quantified using ImageJ software Image J (v.2.9.0). scRNAseq data were collected using DNBSEQ-G400RS instrument (MGI).
Data analysis	Statistical data were analyzed by R (v.3.6.1). scRNAseq data were processed using Cell Ranger software (v6.0.2), and analyzed by R (v.3.6.1) with Seurat (v.4), and Python (v.3.8.2) with Scanpy (v.1.9.1).

For manuscripts utilizing custom algorithms or software that are central to the research but not yet described in published literature, software must be made available to editors and reviewers. We strongly encourage code deposition in a community repository (e.g. GitHub). See the Nature Portfolio guidelines for submitting code & software for further information.

Data

Policy information about availability of data

All manuscripts must include a data availability statement. This statement should provide the following information, where applicable:

- Accession codes, unique identifiers, or web links for publicly available datasets
- A description of any restrictions on data availability
- For clinical datasets or third party data, please ensure that the statement adheres to our policy

All scRNA-seq datasets have been deposited in the National Center for Biotechnology Information Gene Expression Omnibus (GSE 190043). All data underlying the findings are fully available within the paper and its Supplementary data. Correspondence and requests for materials should be addressed to Yoshiakira Kanai (ykanai@g.ecc.u-tokyo.ac.jp). Source Data are provided with this paper.

Field-specific reporting

Please select the one below that is the best fit for your research. If you are not sure, read the appropriate sections before making your selection.

☒ Life sciences ☐ Behavioural & social sciences ☐ Ecological, evolutionary & environmental sciences

For a reference copy of the document with all sections, see nature.com/documents/nr-reporting-summary-flat.pdf

Life sciences study design

All studies must disclose on these points even when the disclosure is negative.

Sample size	Throughout this study, "n" refers to the number of animals. Exact n values are provided in the Methods section in the manuscript. At least three distinct animals were used for each analysis. We used as many animal as possible available for each experiment.
Data exclusions	No data were excluded in this study.
Replication	To ensure the reproducibility, all the experiments were repeated for three times (technical replicate) with similar results. All the experiments were performed with at least three distinct animals (biological replicates) with similar results except for scRNAseq. For scRNAseq, data were obtained from two independent biological replicates.
Randomization	SF1Cre:Sox17flox/flox mice were allocated to Sox17-cKO group (experimental group), while their Sox17flox/flox littermate control was allocated to the Control group. For the analysis of mutant mice, randomization was not feasible since genotypes of the mice needed to be determined.
Blinding	Blinding was applicable in this study since the authors needed to be aware of genotypes of the mice to perform all analysis.

Reporting for specific materials, systems and methods

We require information from authors about some types of materials, experimental systems and methods used in many studies. Here, indicate whether each material, system or method listed is relevant to your study. If you are not sure if a list item applies to your research, read the appropriate section before selecting a response.

Materials & experimental systems

n/a	Involved in the study	
☐	☒	Antibodies
☒	☐	Eukaryotic cell lines
☒	☐	Palaeontology and archaeology
☐	☒	Animals and other organisms
☒	☐	Human research participants
☒	☐	Clinical data
☒	☐	Dual use research of concern

Methods

n/a	Involved in the study	
☒	☐	ChIP-seq
☒	☐	Flow cytometry
☒	☐	MRI-based neuroimaging

Antibodies

Antibodies used

- primary antibodies [format: host anti-protein (company, catalog number, dilution, lot number & Clone number if available)]
1. mouse monoclonal anti-ace-TUB (Sigma, T6793, 1:200, 059M4876V, clone 6-11B-1)
2. mouse monoclonal anti-aSMA/ACTA2 (Sigma, A5228, 1:500, clone 1A4)
3. goat polyclonal anti-AMH (Santa Cruz, sc6886, 1:200)
4. mouse monoclonal anti-CDH1 (BD Transduction lab, 610181, 1:400, 8082613, clone 36/E-Cadherin)
5. goad polyclonal anti-c-KIT (R&D systems, AF1356, 1:200, IEO0215111)
6. rabbit monoclonal anti-EPCAM (Abcam, ab32392, 1:100, clone E144)
7. goat polyclonal anti-GATA4 (Santa Cruz, sc1237, 1:200, J2015)
8. mouse monoclonal anti-GATA4 (Santa Cruz, sc25310, 1:100, I2321, clone G-4)
9. rabbit polyclonal anti-GFP (MBL, 598, 1:200, 078)
10. goat polyclonal GFRa1 (R&D systems, AF560, 1:100, BQE0520031)
11. rabbit polyclonal anti-HSP70 (Abcam, ab79852, 1:1000, GR281565-44)
12. rabbit polyclonal anti-KI67 (Abcam, ab15580, 1:400, GR3375617-1)
13. rabbit monoclonal anti-KRT8 (Abcam, ab53280, 1:200, GR3241174-12, clone EP1628Y)
14. rabbit polyclonal LAMININ (Abcam, ab11575, 1:200, GR3398522-1)
15. rabbit monoclonal anti-p-AKT (Cell Signaling, 4060, 1:100, clone D9E)
16. mouse monoclonal anti-PAX8 (Abcam, ab53490, 1:100, GR3235753-6, clone PAX8R1)
17. rat monoclonal PECAM1 (Invitrogen, 14-0311-82, 1:100, clone 390)

18. rabbit polyclonal anti-PLZF (Santa Cruz, sc22839, 1:200, G1414)
19. mouse monoclonal SCP3 (Santa Cruz, sc74569, 1:500, L0313, clone D-1)
20. goat polyclonal anti-SOX17 (R&D systems, AF1924, 1:200)
21. rabbit polyclonal anti-SOX9 (Millipore, AB5535, 1:400, 3836442)
22. rabbit polyclonal anti-STAR (Cell Signaling, 8449, 1:100)
23. rabbit polyclonal anti-VASA/MVH (Abcam, ab13840, 1:10000, GR3288578-2)

- secondary antibodies [format: host anti-protein (company, catalog number, dilution, lot number if available)]:
24. chicken anti-mouse Alexa 488 conjugated (Invitrogen, A-21200, 1597055)
25. chicken anti-rabbit Alexa 488 conjugated (Invitrogen, A-21441, 1304742)
26. donkey anti-goat Alexa 488 conjugated (Invitrogen, A-11058, 1445994)
27. goat anti-mouse Alexa 594 conjugated (Invitrogen, A-11032, 2301112)
28. chicken anti-rabbit Alexa 594 conjugate (Invitrogen, A-21442, 1827987)
29. donkey anti-goat Alexa 594 conjugate (Invitrogen, A-11058, 2306782)
30. donkey anti-rabbit Alexa 680 (Invitrogen, A-10043)
31. horse anti-mouse IgG biotinylated (Vector Laboratories, BA-2001)
32. goat anti-rabbit IgG biotinylated (Vector Laboratories, BA-1000, ZF0809)
33. rabbit anti-goat IgG biotinylated (Vector Laboratories, BA-5000, ZF0306).

Validation

All the antibodies used in this study are commercially available, validated by the manufactures or by our laboratory:
1. Anti-ace-TUB (https://www.sigmaaldrich.com/JN/en/product/sigma/t6793)
2. Anti-aSMA/ACTA2 (https://www.sigmaaldrich.com/JN/en/product/sigma/a5228)
3. Anti-AMH (https://www.citeab.com/antibodies/814608-sc-6886-mis-antibody-c-20)
4. Anti-CDH1 (https://www.bdbiosciences.com/en-eu/products/reagents/microscopy-imaging-reagents/immunofluorescence-reagents/purified-mouse-anti-e-cadherin.610181)
5. Ant-c-KIT (https://www.rndsystems.com/products/human-mouse-cd117-c-kit-antibody_af1356)
6. Anti-EPCAM (https://www.abcam.com/epcam-antibody-e144-ab32392.html)
7. Anti-GATA4 (https://www.scbt.com/p/gata-4-antibody-c-20)
8. Anti-GATA4 (https://www.scbt.com/p/gata-4-antibody-g-4)
9. Anti-GFP (https://ruo.mbl.co.jp/bio/dtl/A/?pcd=598)
10. Anti-GFRa1 (https://www.rndsystems.com/products/rat-gfr-alpha-1-gdnf-r-alpha-1-antibody_af560)
11. Anti-HSP70 (https://www.abcam.com/hsp70-antibody-ab79852.html)
12. Anti-KI67 (https://www.abcam.com/ki67-antibody-ab15580.html)
13. Anti-KRT8 (https://www.abcam.com/cytokeratin-8-antibody-ep1628y-cytoskeleton-marker-ab53280.html)
14. Anti-LAMININ (https://www.abcam.com/laminin-antibody-ab11575.html)
15. Anti-p-AKT (https://en.cellsignal.jp/products/primary-antibodies/phospho-akt-ser473-d9e-xp-rabbit-mab/4060)
16. Anti-PAX8 (https://www.abcam.com/pax8-antibody-pax8r1-ab53490.html)
17. Anti-PECAM1 (https://www.thermofisher.com/antibody/product/CD31-PECAM-1-Antibody-clone-390-Monoclonal/14-0311-82)
18. Anti-PLZF (https://www.scbt.com/p/plzf-antibody-h-300)
19. Anti-SCP3 (https://www.scbt.com/p/scp-3-antibody-d-1)
20. Anti-SOX17 (https://www.rndsystems.com/products/human-sox17-antibody_af1924)
21. Anti-SOX9 (https://www.merckmillipore.com/JP/ja/product/Anti-Sox9-Antibody,MM_NF-AB5535?ReferrerURL=https%3A%2F%2Fwww.google.com%2F)
22. Anti-STAR (https://en.cellsignal.jp/products/primary-antibodies/star-d10h12-xp-rabbit-mab/8449)
23. Anti-VASA/MVH (https://www.abcam.com/ddx4--mvh-antibody-ab13840.html)
24. Anti-mouse Alexa 488 conjugated (lhttps://www.thermofisher.com/antibody/product/Chicken-anti-Mouse-IgG-H-L-Cross-Adsorbed-Secondary-Antibody-Polyclonal/A-21200)
25. Anti-rabbit Alexa 488 conjugated (https://www.thermofisher.com/antibody/product/Chicken-anti-Rabbit-IgG-H-L-Cross-Adsorbed-Secondary-Antibody-Polyclonal/A-21441)
26. Anti-goat Alexa 488 conjugated (https://www.thermofisher.com/antibody/product/Donkey-anti-Goat-IgG-H-L-Cross-Adsorbed-Secondary-Antibody-Polyclonal/A-11058)
27. Anti-mouse Alexa 594 conjugated (https://www.thermofisher.com/antibody/product/Goat-anti-Mouse-IgG-H-L-Highly-Cross-Adsorbed-Secondary-Antibody-Polyclonal/A-11032)
28. Anti-rabbit Alexa 594 conjugate (lhttps://www.thermofisher.com/antibody/product/Chicken-anti-Rabbit-IgG-H-L-Cross-Adsorbed-Secondary-Antibody-Polyclonal/A-21442)
29. Anti-goat Alexa 594 conjugate (https://www.thermofisher.com/antibody/product/Donkey-anti-Goat-IgG-H-L-Cross-Adsorbed-Secondary-Antibody-Polyclonal/A-11058)
30. Anti-rabbit Alexa 680 (https://www.thermofisher.com/antibody/product/Donkey-anti-Rabbit-IgG-H-L-Highly-Cross-Adsorbed-Secondary-Antibody-Polyclonal/A10043)
31. Anti-mouse IgG biotinylated (https://vectorlabs.com/products/antibodies/biotinylated-horse-anti-mouse-igg-rat-adsorbed)
32. Anti-rabbit IgG biotinylated (https://vectorlabs.com/products/antibodies/biotinylated-goat-anti-rabbit-igg)
33. Anti-goat IgG biotinylated (https://vectorlabs.com/products/antibodies/biotinylated-rabbit-anti-goat-igg).

Animals and other organisms

Policy information about studies involving animals; ARRIVE guidelines recommended for reporting animal research

Laboratory animals

Housing condition: Animals were provided with water and commercial laboratory mouse chow ad libitum and were housed under controlled lighting conditions (daily light from 08:00 to 20:00) with temperatures of 18-23°C and 40-60% humidity.
Species: Mus musculus

	Strain: C57B6/BL, Nr5a1Cre, SF1Cre:Sox17 flox/flox, Sox17flox/flox, AMH-Treck, WBB6F1/Kit-KitW/KitW-v/Slc (W/Wv), SF1Cre:ROSA26tdTomato, Sox17-eGFP knock-in mice.<br>Sex: Since it is a study focused on testis, only males were used for data collection,<br>Age: E12.5-18,5, 1, 2, 3, 4, 8-week-old, 11-week-old
Wild animals	No wild animals were used in this study.
Field-collected samples	No field-collected samples were used in this study.
Ethics oversight	All animal experiments were performed following the Guidelines for Animal Use and Experimentation at the University of Tokyo, and all experimental procedures performed herein were approved by the Institutional Animal Care and Use Committee, Graduate School of Agricultural and Life Sciences, University of Tokyo (approval IDs: P13-762 and P13-764).

Note that full information on the approval of the study protocol must also be provided in the manuscript.
